# Identification and validation of ANXA3 and SOCS3 as biomarkers for acute myocardial infarction related to sphingolipid metabolism

**DOI:** 10.1186/s41065-025-00515-3

**Published:** 2025-08-04

**Authors:** Ling Sun, Lingyan He, Hai-Hua Pan, Chang-Lin Zhai

**Affiliations:** 1 Zhejiang Chinese Medical University, Hangzhou City, Zhejiang Province China; 2 Department of Cardiology, The First Hospital of Jiaxing Affiliated Hospital of Jiaxing University, 1882 South Zhonghuan Road, Nanhu, Jiaxing, Zhejiang 314001 China

**Keywords:** Acute myocardial infarction, Sphingolipid metabolism, Machine learning, Immune microenvironment, Single cell RNA analysis

## Abstract

**Background:**

Sphingolipid metabolism (SM) is linked to acute myocardial infarction (AMI), but its role remains unclear. This study explored SM-related genes (SMRGs) in AMI to support clinical diagnosis.

**Methods:**

We analyzed datasets GSE48060 and GSE123342 to identify differentially expressed genes (DEGs) and key module genes. Protein-protein interaction (PPI) network analysis and machine learning were used to screen potential biomarkers, which were validated via receiver operating characteristic (ROC) curves and expression assessment. Further analyses included artificial neural networks (ANN), enrichment analysis, immune infiltration, drug prediction, and molecular docking. Single-cell RNA sequencing (scRNA-seq) identified key cell types and their functions. Biomarkers were validated via reverse transcription quantitative polymerase chain reaction (RT-qPCR).

**Results:**

Intersection of 95 DEGs and 2,196 module genes yielded 20 genes, with *ANXA3* and *SOCS3* identified as biomarkers. The ANN model showed superior diagnostic performance compared to individual markers. Biomarkers were enriched in the toll-like receptor (TLR) signaling pathway. Immune infiltration analysis revealed differences in five immune cell types between AMI and control groups. *ANXA3* correlated positively with neutrophils and negatively with resting memory CD4 T cells. Drugs targeting *ANXA3* included ethanolamine, difluocortolone, and fluocinolone acetonide, with strong binding affinity. scRNA-seq identified B cells and monocytes as key cells; *ANXA3* and *SOCS3* expression increased during monocyte differentiation before decreasing, while B cells showed no significant changes.

**Conclusion:**

*ANXA3* and *SOCS3* were identified as SM-related biomarkers in AMI, providing insights for clinical diagnosis.

**Supplementary Information:**

The online version contains supplementary material available at 10.1186/s41065-025-00515-3.

## Introduction

Acute myocardial infarction (AMI) is a severe cardiac condition characterized by chest pain, difficulty breathing, and a high mortality rate. Epidemiological studies show that AMI is one of the leading causes of death worldwide, with a particularly high prevalence in industrialized countries [1]. The disease pathogenesis involves ischemia and necrosis of myocardial tissue resulting from coronary artery blockage, affecting individuals with underlying conditions such as hypertension, hyperlipidemia, and diabetes. Early detection and prompt intervention are critical, given AMI’s poor short-term prognosis. Although cardiac biomarkers such as creatine kinase-MB (CK-MB) and cardiac troponin (cTn) are widely used for early diagnosis, issues such as false-positives results remain a challenge. Furthermore, AMI leads to irreversible myocardial cell loss, which can trigger left ventricular remodeling and progression to heart failure. Despite advances in pharmacological treatments and percutaneous coronary interventions, improving patient prognosis remains a persistent challenge. A deeper understanding of AMI’s complex pathophysiology and the identification of novel biomarkers are essential for advancing therapeutic strategies and improving patient outcomes [2]. Metabolomics, with sphingolipids as a focus of research, has emerged over the past decade as a critical tool for understanding the onset and progression of AMI [3]. Sphingolipid metabolism (SM) is closely related to the regulation of both apoptosis and cell proliferation, making it a foundational aspect of the physiopathology of various diseases. Additionally, SM is crucial in cell signaling pathways. Research has shown that sphingomyelin and its metabolites contribute to a complex signaling network that influences multiple cellular processes. The activation of sphingomyelinase, the enzyme catalyzing the first step in SM turnover, has been linked to cellular differentiation [4, 5]. Moreover, the sphingomyelinase pathway is closely associated with apoptosis in response to environmental stress, with acid sphingomyelinase activation being particularly important for apoptotic responses [6]. Beyond heart disease, SM plays an important role in a number of other conditions by regulating cell growth, differentiation and programmed cell death. Key metabolites of SM, including ceramide and sphingosine, have been identified as playing pivotal roles in cardiovascular diseases [7]. These results establish a theoretical basis for additional exploration of the function of SM metabolism in AMI, presenting opportunities for novel treatment approaches [8, 9]. While SM and its metabolic products are thought to be involved in AMI pathogenesis, the precise function of SM-related genes (SMRGs) in AMI remains to be fully elucidated.

ScRNA-seq technology enables the extraction and analysis of genetic information the level of a single cell and includes the amplification of the genome and transcriptome as well as high-throughput sequencing. This method demonstrates that RNA transcripts within individual cells possess diversity and complexity, shedding light on the distinct constituents of cell types and their specific capabilities within tissues, organs, or the whole organism. scRNA-seq has become an advanced tool for understanding cellular heterogeneity and tissue architecture [10]. In cardiovascular research, this technology has proven pivotal. For example, it has been used to explore cellular reprogramming and communication in atherosclerosis, providing novel insights into plaque vulnerability [11]. Moreover, scRNA-seq has been used to unravel the heterogeneity of cardiac fibroblasts, key players in the pathogenesis of heart failure, highlighting their role in MI and their potential as therapeutic targets [12]. Furthermore, scRNA-seq has been crucial in examining immune cell infiltration dynamics post-AMI, unveiling the intricate interplay between immune responses and cardiac tissue repair [13]. These studies highlight the importance of scRNA-seq in elucidating the cellular and molecular mechanisms underlying cardiovascular diseases, including AMI, and in identifying new therapeutic strategies. With ongoing technological advancements and decreasing costs, scRNA-seq is increasingly applied in fields such as genomics, developmental biology, reproductive biology, and evolutionary biology [14–17].

In the present study, datasets from two public databases, GSE48060 and GSE123342, were analysed. Subsequently, differential gene expression analysis and WGCNA were conducted, identifying candidate genes linked to SMRGs in AMI. Networks PPI, along with machine learning techniques, validation of expression, and ROC curves, were additionally employed to identify possible biomarkers and explore their biological pathways and connections with the immune microenvironment. This research revealed the diagnostic value of SMRGs in AMI and explored their underlying molecular regulatory mechanisms, providing theoretical basis for the clinical development of innovative therapeutic approaches and drugs.

## Materials and methods

### Data extraction

The datasets GSE48060 (platform: GPL570), GSE123342 (platform: GPL17586), and GSE269269 (platform: GPL24676) were sourced from the Gene Expression Omnibus (GEO) database (http://www.ncbi.nlm.nih.gov/geo/). The training set GSE48060 includes blood samples from 31 AMI patients and 21 controls. The validation set, GSE123342, included 192 peripheral blood samples from 67 patients with AMI and 22 control samples and GSE60993, included 24 peripheral blood samples from 17 patients with AMI and 7 control samples. All samples were peripheral blood samples. Additionally, GSE269269 was a scRNA-seq dataset comprising peripheral blood mononuclear cell samples from 5 patients with AMI with PR and 5 without plaque rupture (NPR). A total of 97 SMRGs were obtained from the published literature [18].

### Differential expression analysis

Differential expression analysis of the GSE48060 dataset was performed using the limma package (v3.54.0) [19] to identify DEGs between the AMI and controls, with screening criteria of *p* < 0.05 and |log_2_ FoldChange(FC)| >0.5. The ggplot2 package (v3.4.1) [20] was used to generate volcano plots for visualizing DEG expression. Additionally, creating heatmaps with pheatmap package (v1.0.12) [21] to show the expression patterns of the top 10 up- and down-regulated DEGs.

### Weighted gene co-expression network analysis (WGCNA)

Using the 97 SMRGs, the ssGSEA algorithm in the GSVA package (v1.38.2) [22] was applied to calculate the SMRGs score for AMI samples in GSE48060. WGCNA was performed using the WGCNA package (v1.72-1) [23] to identify modules most associated with SM, using the SMRGs score as a feature. To identify potential outliers, construct a hierarchical clustering tree using AMI samples within the training set. Determine the ideal soft threshold, assure interplay between genes conform to a scale-free distribution. The genes are clustered into different modules of at least 100 genes each by hybridization dynamic tree chopping algorithm. Pearson correlation analysis was conducted to identify the module most strongly associated with SM (correlation coefficient [24] > 0.5, *p* < 0.05). For further analysis, the genes within these modules were designated as key module genes.

### Identification of candidate genes

Crossover genes were identified by overlapping DEGs and key module genes. The package ggvenn (v0.1.9) was used [25]. Enrichment analysis of crossover genes using the ClusterProfiler package (v4.7.1) [26] and the org.Hs.eg.db package (v3.16.0) [27] to explore GO and KEGG pathways, focusing on BP, MF and CC (*p* < 0.05). PPI networks were built to explore interactions between crossover genes by STRING database (https://string-db.org). Finally, the closeness, ecCentricity, and degree computing method in the CytoHubba plugin were used in the intersection genes, select the top 10 genes, and candidate genes were identified through their overlap.

### Qualification of biomarkers

Based on candidate genes support vector machine recursive feature elimination (SVM-RFE) and XGBoost analyses were conducted using the caret package (v6.0-93) [28] and the xgboost package (v1.7.3.1) [29], respectively, to identify key features. The resulting featured genes from both analyses were then intersected to define the candidate biomarkers. To evaluate the expression of these biomarkers, the Wilcoxon test was performed on the GSE48060 and GSE123342 datasets, comparing AMI and control groups (*p* < 0.05). ROC curves were plotted using the pROC package (v1.18.0) to further assess the diagnostic ability of these biomarkers [30]. In both datasets, those with consistent expression trends, significant differences, and AUC values above 0.7 were considered as potential biomarkers for subsequent analyses. The GSE60993 was used for biomarker validation. Additionally, the ROC curves for traditional AML biomarkers (MB and cTn, including TNNI3, TNNT2, and TNNC1) were plotted in the training set GSE48060 and the validation set GSE60993, and their diagnostic abilities were compared to those of potential biomarkers using AUC values.

### Construction of artificial neural network (ANN)

An ANN model was then constructed based on the identified biomarkers, using the neuralnet package (v1.18.0) [30]. Data preprocessing was used to standardise the data. This was followed by min-max normalisation before training the neural network. The data were standardized for both maximum and minimum values, and a single hidden layer was established, selecting the number of neurons to be positioned between the input and output layers. To avoid overfitting and enhance the reliability of model evaluation, 5-fold cross-validation is employed. This method enables the assessment of the model’s performance on different data subsets, thereby providing a more accurate reflection of the model’s generalization ability. The ANN model built on the training set was tested for performance on the validation set. The ANN model was configured with 2 hidden neurons. The significance of each biomarker in the model’s prediction was computed and visualized. Additionally, the function of ANN model was assessed using a ROC curve, while Spearman relationship analysis of biomarkers using a correlation graph software package (v0.92) [27]. To mitigate the risk of overfitting, logistic regression (LR) analysis was employed, utilizing the sigmoid function to map the linear model output (wTx) into the [0,1] interval, thereby endowing it with probabilistic interpretation. To enhance the reliability of the results, ANN was iteratively constructed and validated on the verification set, with performance further assessed through ROC analysis.

### Functional analysis of biomarkers

Genes associated with biomarkers function were predicted using the GENEMANIA database (http://genemania.org/). GSEA was then performed on the biomarkers using the KEGG background (c2.cp.kegg.v2023.2.Hs.symbols.gmt) to identify the signaling pathways, which the biomarkers were involved. The smples from the GSE48060 dataset were divided into a high expression group and a low expression group based on the median expression value of the biomarkers. Differential expression analysis was conducted using the limma package (v3.54.0)19. Genes were sorted according to log_2_FC, and GSEA was performed with the criteria p.adj < 0.05 and |normalized enrichment score (NES)| ≥ 1.

### Immune infiltration analysis

Investigate the connection between AMI and the immune micro-environment, we conducted an immune infiltration analysis using the GSE48060 dataset. The CIBERSORT algorithm [31] was used to assess the presence of 22 different immune cell types in all samples. It employed linear support vector regression (SVR) deconvolution technology to match mixed gene expression data with the reference signature matrix (LM22) of 22 immune cell subsets, thereby calculating the proportion of each cell type. Immune cells with an infiltration richness zero in over 75% of the samples were excluded, and the remaining immune cells were analyzed. The level of immune cell infiltration in AMI and control groups was assessed using the Wilcoxon signed rank sum test. Furthermore, differential immune cells and the correlation partitioning between these immune cells were analyzed using the correlation plot package (v0.92), |cor| >0.3, p < for 0.05.

### Construction of TF-miRNA-mRNA network

Study the molecular mechanisms regulating the biomarkers, the miRNet database (https://www.mirnet.ca/miRNet/home.xhtml) was used to predict microRNAs (miRNAs) targeting the biomarkers. Subsequently, the NetworkAnalyst database (https://www.networkanalyst.ca/) was employed to predict TFs targeting the biomarkers. The TF-miRNA-mRNA network was then built using Cytoscape software (v3.8.2) [32].

### Drug prediction and molecular docking

To identify potential targeted drugs, the DGidb (https://dgidb.org/) was used to predict small molecule drugs targeting the biomarkers. Additionally, Cytoscape software was used to illustrate the associations between the biomarkers and the small-molecule drugs. To explore the binding affinity between biomarkers and the active ingredients of small molecule drugs, molecular docking. The PDB files for the biomarkers index from the PDB (https://www1.rcsb.org/). Additionally, the 3D structural data of the active components in SDF format were derived from the PubChem Substance database hosted by NCBI. The most effective proteins and active ingredients were identified using CB-Dock, and the results were visualized using PyMOL. A docking score below − 5 kcal/mol was regarded as evidence of significant binding affinity between the compound and the target [33].

### Molecular dynamics simulation

After exploring the complex interactions between biological markers and specific compounds, a state-of-the-art 100-nanosecond (ns) MD simulation was conducted to assess the steadiness of the post-binding complexes. Utilizing the advanced capabilities of the NAMD software package [34], the biological environment was simulated with high precision using a water model, ensuring that the simulation conditions closely reflected those of biological systems. To quantitatively evaluate protein structure stability throughout the simulation, the root-mean-square deviation (RMSD) metric was employed. RMSD effectively captured atomic fluctuations over time, offering a reliable measure of system stability during the dynamics. By comparing the RMSD values before and after the simulation for the biological marker-compound complexes, the structural integrity and resilience of the complexes were assessed. This foundational analysis provided critical insights that underpin future drug design initiatives and biological marker identification, offering a robust scientific framework for these areas of research.

Molecular docking was first used to simulate the receptor-compound binding model, with binding energy calculations followed by molecular dynamics simulations to verify model stability. The system was prepared by defining simulation boxes through VMD software, positioning the complexes in a cubic box with periodic boundaries, and utilizing the TIP3P water model. The system charge was then neutralized by adding counter ions *via* a 0.15 mol/L NaCl solution [35, 36]. The pre-equilibration process, carried out using the NAMD software package, employed the MMFF force field for the ligand and the CHARMM force field for the receptor. The simulation was performed at 310 K, involving a 200 ps energy minimization phase with a convergence threshold of 5000 kJ/mol/nm [37]. Finally, a 10-ns molecular dynamics simulation was performed, with data recorded every 1 ps.

### scRNA-seq data analysis

Single cells from the GSE269269 dataset were processed using the Seurat package (v 5.1.0) [38], applying filters based on gene expression (> 3), feature count (200 < nFeature_RNA < 500), and mitochondrial gene percentage (< 10%). Data normalization was performed using the NormalizeData function, followed by identification of the top 2000 highly variable genes (HVGs) with large coefficients of variation across cells using the FindVariableFeatures function. The LabelPoint tool was utilized to display the outcomes, emphasizing the top 10 genes with the highest variability. Subsequent normalization of all data was performed using the ScaleData function, followed by dimensionality reduction *via* principal component analysis (PCA), and an elbow plot was generated using the ElbowPlot function. Cell clustering was then performed using the FindNeighbors and FindClusters functions (resolution = 0.4), and cell population clustering was visualised using the Uniform Manifold Approximation and Projection (UMAP) algorithm. Cell clusters were annotated, and marker genes were identified based on literature for AMI single-cell studies [39, 40]. The distribution of cell types in PR and NPR individuals from the GSE269269 dataset was visualized, with key cells distinguished by their elevated or reduced expression of marker genes. The CellChat software package (version 1.6.1) was used to analyze intercellular communication for annotated cell types [41]. To investigate gene expression changes during cell state transitions in the GSE269269 dataset, key cells underwent dimensionality reduction and clustering using the RunPCA, FindNeighbors, and FindClusters functions. These subpopulations were further analyzed for cell trajectory using the Monole package (v 2.30.1) [42].

### Expression validation of biomarkers

Blood samples were taken from five AMI patients in Jiaxing First Hospital of, with five healthy individuals serving as controls. Total RNA was extracted from these samples for reverse transcription-polymerase chain reaction (RT-qPCR) analysis. This research was approved by the Ethics Committee of the First Hospital of Jiaxing, Affiliated Hospital of Jiaxing University (2022-LY-002), and all participants provided informed consent. The validation of biomarker expression was conducted using RT-qPCR. Total RNA was isolated from 10 samples by referring to the TRIzol reagent (Ambion, Austin, USA) instructions. Refer to the cDNA Synthesis Kit (Service Wuhan, China) instructions to generate cDNA from total RNA. RT-qPCR use 2xUniversal Blue SYBR Green qPCR Master Mix (Servicebio, Wuhan, China) with GAPDH as the internal reference gene. Primer sequences for RT-qPCR are listed in Supplementary material 1. 2^−ΔΔCt^ method to calculate relative gene expression [43].

### Statistical analysis

Data analytics conducted using R software (v 4.2.1), and group differences were assessed using the Wilcoxon test. A *p*-value < 0.05 was considered statistically significant.

## Results

### Analysis of AMI differential genes and SM-related module genes

**Fig. 1:**
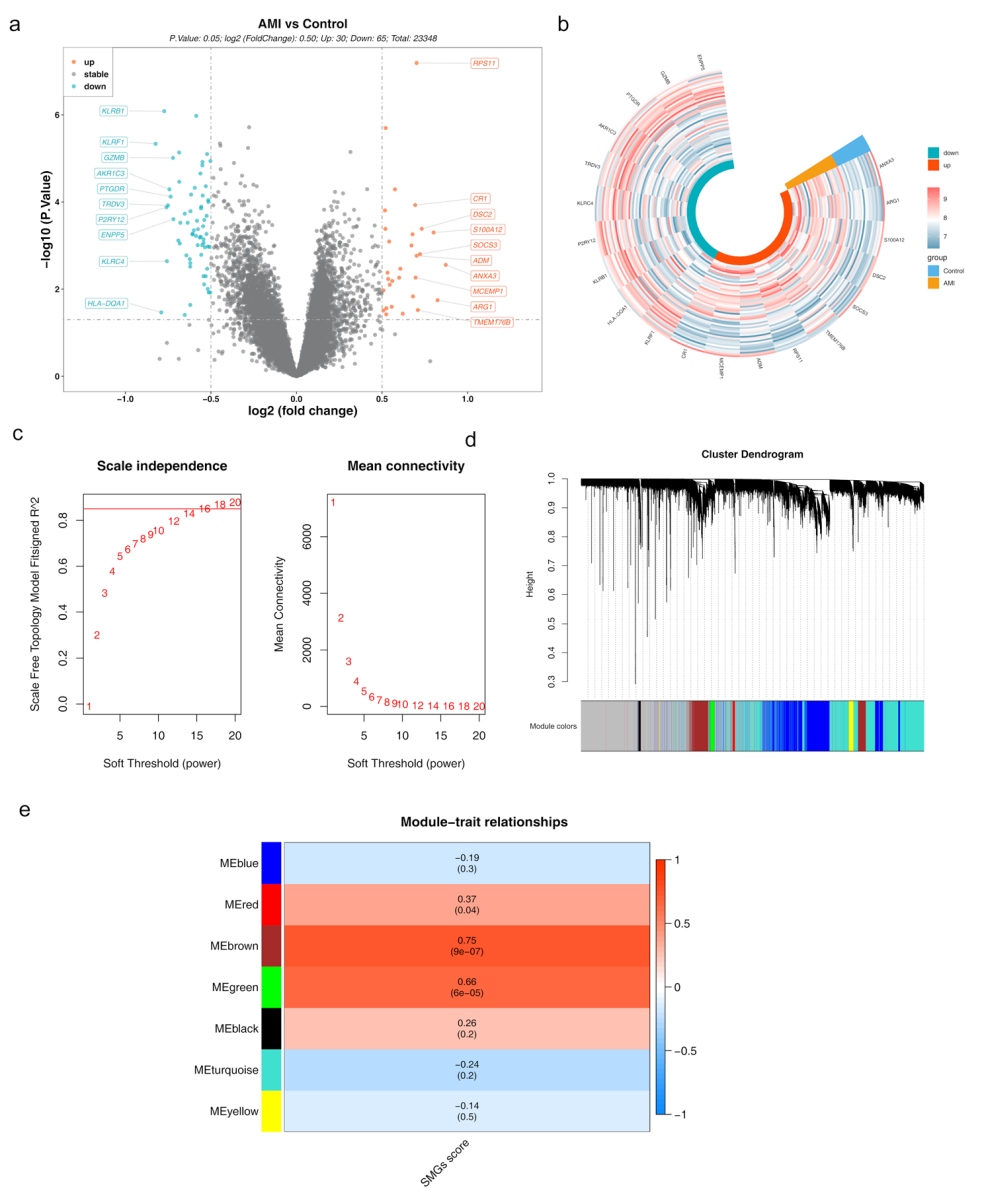
Differential gene expression analysis and WGCNA in AMI (**a**) Volcano plot depicting differentially expressed genes (DEGs) between patients with AMI and control samples, highlighting upregulated (red) and downregulated (blue) genes. (**b**) Heatmap displaying the number of upregulated versus downregulated DEGs in the AMI dataset. (**c**) Plot showing the selection of the soft-thresholding power for constructing a scale-free network in WGCNA. (**d**) Cluster dendrogram from WGCNA identifying gene modules associated with sphingolipid metabolism in AMI. (**e**) Heatmap illustrating the correlation between identified gene modules and SMRGs

95 DEGs between the AMI and control groups were identified in the GSE48060 dataset, with 30 DEGs upregulated and 65 DEGs downregulated (Fig. 1a-b). WGCNA was performed to identify the gene modules most associated with SM. Hierarchical clustering analysis revealed no outliers among the AMI samples in the GSE48060 dataset (Supplementary material 2). Based on the optimal soft threshold of 16 (Fig. 1c), seven modules were identified through cluster analysis (Fig. 1d). Analysis demonstrated, the MEbrown (cor = 0.75, *p* = 9 × 10^− 7^) and MEgreen (cor = 0.66, *p* = 6 × 10^− 5^) modules exhibited the most significant positive correlation with the SMRGs score. These two modules, containing 2,196 genes, were selected for subsequent analysis (Fig. 1e).

### Candidate genes were recognized in AMI

**Fig. 2:**
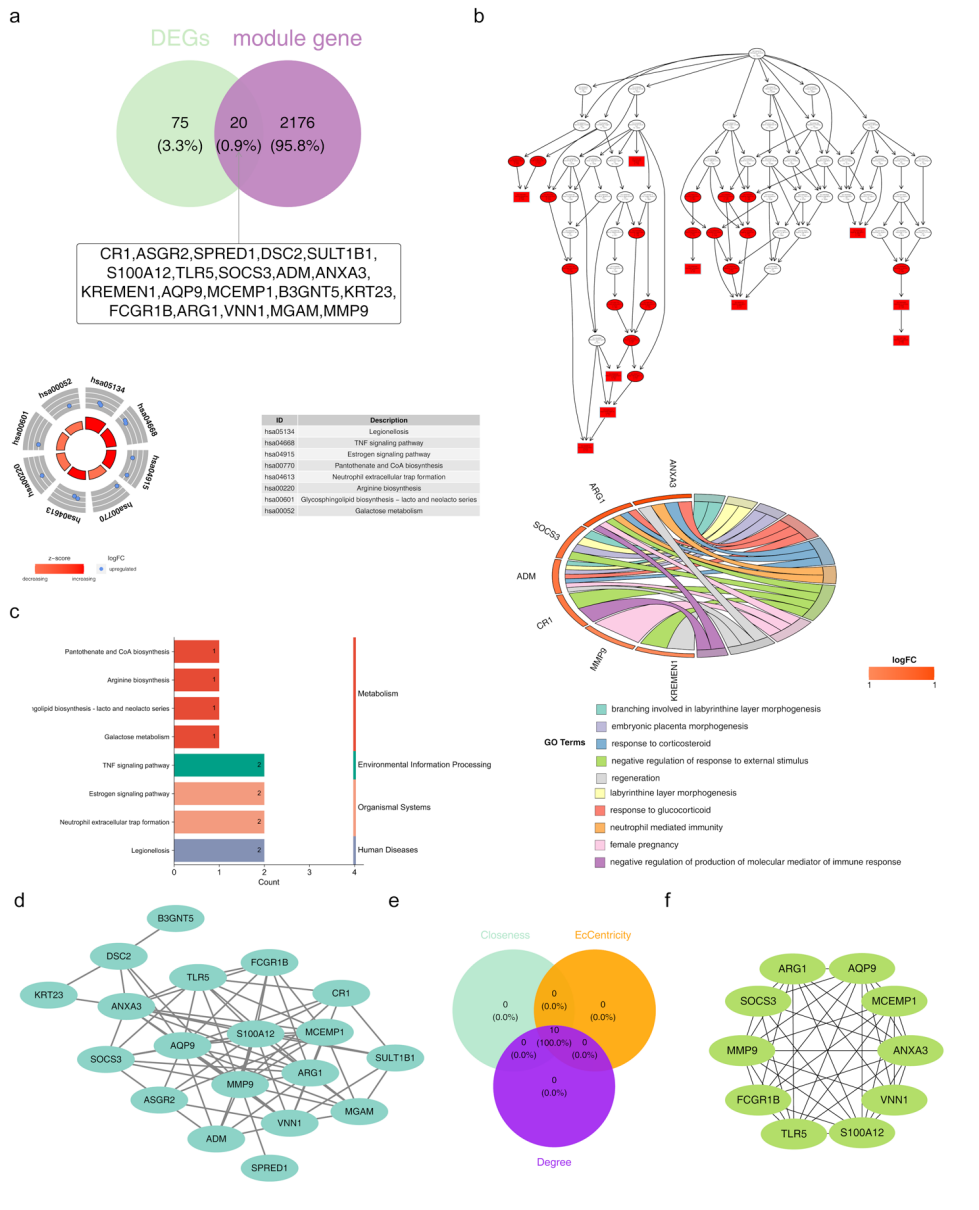
Identification and functional analysis of candidate genes (**a**) Venn diagram depicting the intersection of DEGs and sphingolipid metabolism-related module genes. (**b**) Chord diagram illustrating GO term enrichment of the intersecting genes, focusing on biological processes, molecular functions, and cellular components. (**c**) Bar graph showing the KEGG pathway enrichment of intersecting genes. (**d**) Protein-protein interaction (PPI) network of the intersecting genes, highlighting significant interactions. (**e**) Identification of top candidate genes using network analysis tools, emphasizing centrality measures. (**f**) Interaction network of top candidate genes, outlining potential regulatory roles in AMI. *p < 0.05, **p < 0.01, ***p < 0.001 represented comparison of AMI with control group

Through the intersection of the 95 DEGs and the 2,196 genes associated with the SM module, a total of 20 genes were found to overlap (Fig. 2a). Enrichment analysis was performed to investigate the common biological functions and pathways associated with these intersection genes. The 20 identified genes showed significant enrichment across 401 GO terms (347 BP, 19 CC, and 35 MF), such as branching in labyrinthine layer morphogenesis, the morphogenesis of the embryonic placenta, the negative regulation of responses to external stimuli, and the immunity mediated by neutrophils. (Fig. 2b). Additionally, these genes were enriched in eight KEGG pathways, including TNF signaling, estrogen signaling, pantothenate and CoA biosynthesis, neutrophil extracellular trap formation, arginine biosynthesis, glycosphingolipid biosynthesis, galactose metabolism, and legionellosis (Fig. 2c). The KEGG pathway enrichment results were primarily categorized into four domains: metabolism, environmental information processing, biological systems, and human diseases. Notably, all candidate genes in these pathways were upregulated, with a z-score > 0, suggesting pathway activation. Furthermore, a PPI network is constructed, with 19 nodes and 72 edges. Within this network, *ANXA3* interacted with multiple genes, including *KRT23*, *DSC2*, *SOCS3*, *AQP9*, *S100A12*, and *TLR5* (Fig. 2d). The CytoHubba plug-in, the top 10 genes ranked by closeness, eccentricity, and degree were intersected to identify 10 key candidate genes: *MMP9*, *AQP9*, *S100A12*,* ANXA3*,* ARG1*,* VNN1*,* MCEMP1*,* TLR5*,* FCGR1B*, and *SOCS3* (Fig. 2e). These 10 candidate genes exhibited significant interactions with one another (Fig. 2f).

### *ANXA3* and *SOCS3* were identified as biomarkers in AMI

**Fig. 3:**
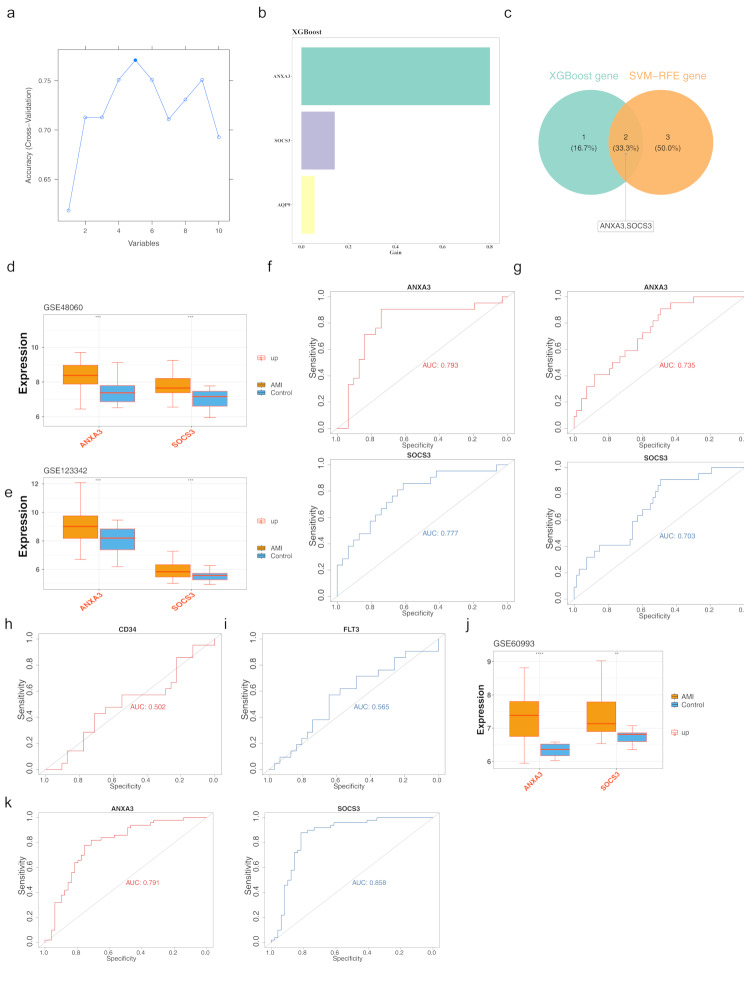
Validation of ANXA3 and SOCS3 as biomarkers (**a**) Feature selection using the SVM-REF algorithm for candidate genes. (**b**) Feature selection via the XGBoost algorithm for candidate genes. (**c**) Venn diagram illustrating the overlap of candidate biomarkers (**d**, **e**) Box plots showing significant differences in expression levels between AMI and control groups (**f**, **g**) AUC values for *ANXA3* and *SOCS3*, validating their efficacy as biomarkers. (**h**, **i**) AUC values for *CD34* and *FLTS.* (**j**) The expression profile of biomarkers in the external validation set, with yellow representing AMI patients and blue representing healthy controls; orange indicates up-regulated expression, blue indicates down-regulated expression, and gray indicates non-significant expression. (**k**) ROC analysis of biomarkers in the validation set, with specificity as the x-axis and sensitivity as the y-axis. The area under the curve represents the AUC value, indicating accuracy (predictive performance)

Two candidate biomarkers, *ANXA3* and *SOCS3*, were selected based on the overlap of five feature genes from the SVM-REF algorithm and three feature genes from the XGBoost algorithm (Fig. 3a-c). In the AMI group of the GSE48060 and GSE123342 datasets, the expression ranges of both *ANXA3* and *SOCS3* were salience higher than those in the controls (Fig. 3d-e). The AUC values for *ANXA3* and *SOCS3* in both datasets exceeded 0.7 (Fig. 3f-g), Moreover the AUC values of traditional AMI diagnostic markers *CD34* and *FLT3* were both less than 0.7 (Fig. 3h-i**)**. Therefore, *ANXA3* and *SOCS3* demonstrate superior diagnostic performance compared to traditional AMI diagnostic markers and are more suitable for further research. The expression levels in the training set and the validation set were consistent, and there were significant differences between the disease group and the control group (Fig. 3j), and the ROC curve showed a certain diagnostic effect (0.9 > AUC > 0.7) (Fig. 3k). Additionally, in the training set GSE48060, the AUC values for ANXA3 and SOCS3 were greater than 0.7, while the AUC values for the traditional biomarkers MB and cTn (TNNI3, TNNT2, TNNC1) were less than 0.7, indicating that the diagnostic ability of the potential biomarkers was relatively superior to that of the traditional biomarkers (Supplementary material 3). In the external validation set GSE60993, the AUC values for ANXA3 and SOCS3 were greater than 0.8, while the AUC values for MB and cTn (TNNI3, TNNT2, TNNC1) were less than 0.8, further confirming the superior diagnostic ability of the potential biomarkers (Supplementary material 4).

### The diagnostic ability of the ANN model for AMI was stable

**Fig. 4:**
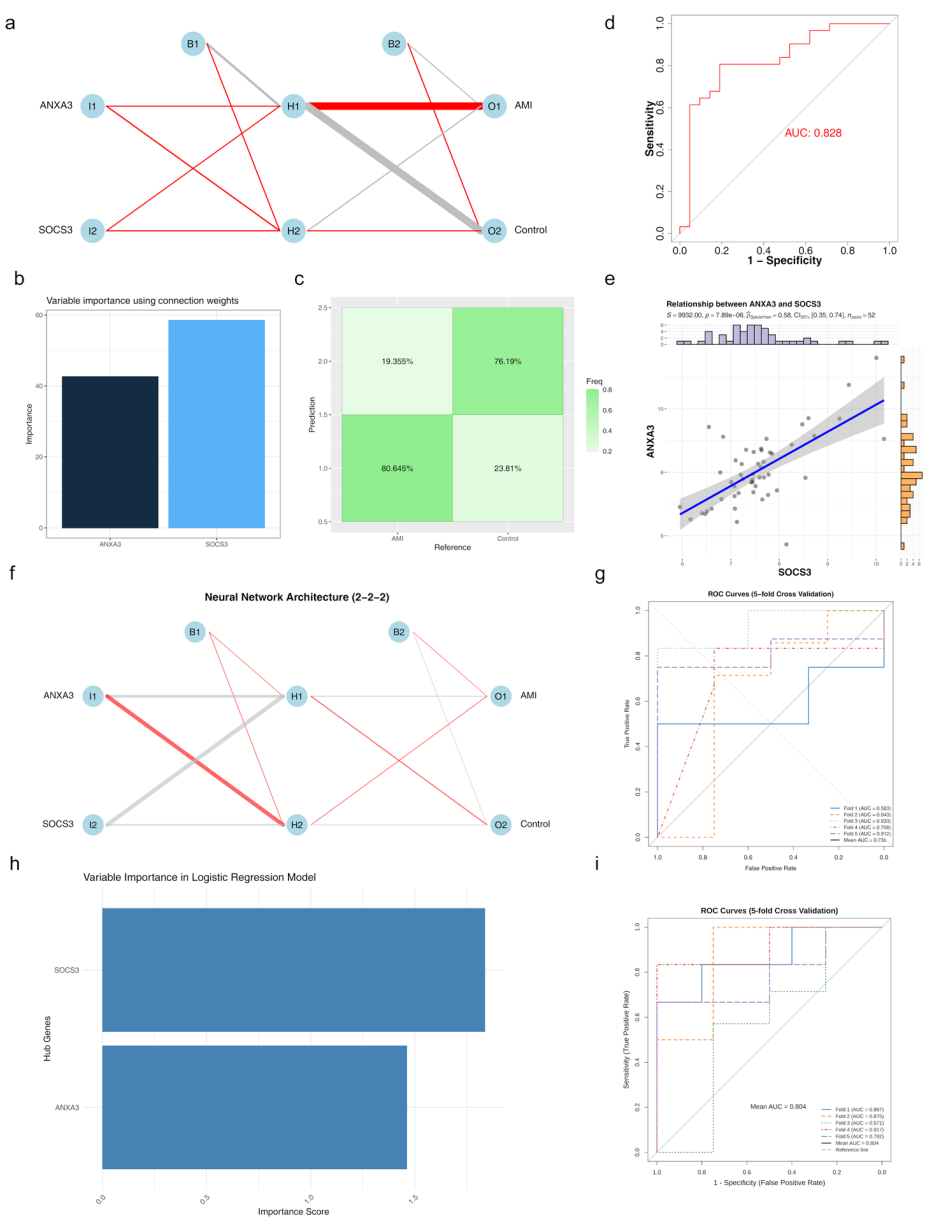
Artificial neural network (ANN) model evaluation (**a**) Schematic representation of the ANN model based on *ANXA3* and *SOCS3*. (**b**) Contribution of each biomarker to the predictive accuracy of the ANN model. (**c**) Confusion matrix illustrating the classification accuracy of the ANN model. (**d**) ROC curve demonstrating the overall diagnostic performance of the ANN model. (**e**) Correlation heatmap among biomarkers, revealing their synergistic effects in AMI diagnosis. (**f**) Visualization of the artificial neural network model. The node labeled “B” here is called the bias unit. The leftmost layer or layer 1 is the input layer, the middle layer or layer 2 is the hidden layer, and the rightmost layer or layer 3 is the output layer. It can be said that the above figure has 2 input units (excluding the bias unit), 2 output units, and 2 hidden units (excluding 1 bias unit). The thickness of the connecting lines represents the weights, which can be considered as the degree of contribution of that variable to the next node. The color of the lines indicates positive or negative contributions, with red representing positive contributions and gray representing negative contributions. (**g**) ROC analysis of the model. (**h**) The importance of independent variables to model prediction outcomes. (**i**) ROC analysis of 5-fold cross-validation

An ANN model was constructed based on the biomarkers *ANXA3* and *SOCS3* (Fig. 4a). *SOCS3* was found to play a more prominent role in the ANN model (Fig. 4b). The confusion matrix indicated that the ANN model achieved an accuracy of 76.19% (Fig. 4c), with an AUC of 0.828 (Fig. 4d). Demonstrating that the model’s diagnostic performance for AMI outperformed that of any individual biomarker. The ANN model built on the training set was tested for performance on the validation set. The results showed that the AUC of the ANN model was 0.916 (Sensitivity: 0.294; Accuracy: 0.5), indicating that the model had certain accuracy and stability (Supplementary material 5). Correlation analysis revealed a significant positive correlation between *ANXA3* and *SOCS3* (*p* < 0.05, cor = 0.58), suggesting a potential synergistic role of these biomarkers in performing related functions (Fig. 4e). A 5-fold cross-validation was conducted to evaluate the performance of the ANN based on *ANXA3* and *SOCS3* (Fig. 4f; Table 1), The ROC curve of the model was plotted using the R package pROC. The average AUC of the ANN model was 0.736, indicating that the model has certain accuracy and stability (Fig. 4g). Two biomarkers from the training set were used for logistic regression analysis to visualize the importance of each independent variable in the model’s prediction results, as shown in Fig. 4h. The most critical variable for classification is *SOCS3*. The model’s performance was evaluated using the ROC curve, as illustrated in Fig. 4I, with an average AUC value of 0.804, indicating that the model has good accuracy and stability.

**Table 1:** Cross-validation performance evaluation metrics

Metric	Mean	SD
AUC	0.736071429	0.139230758
Accuracy	0.727272727	0.150618559
Sensitivity	0.729761905	0.159252612
Specificity	0.75	0.25

### Biomarkers were significantly enriched in the TLR signaling pathway

**Fig. 5:**
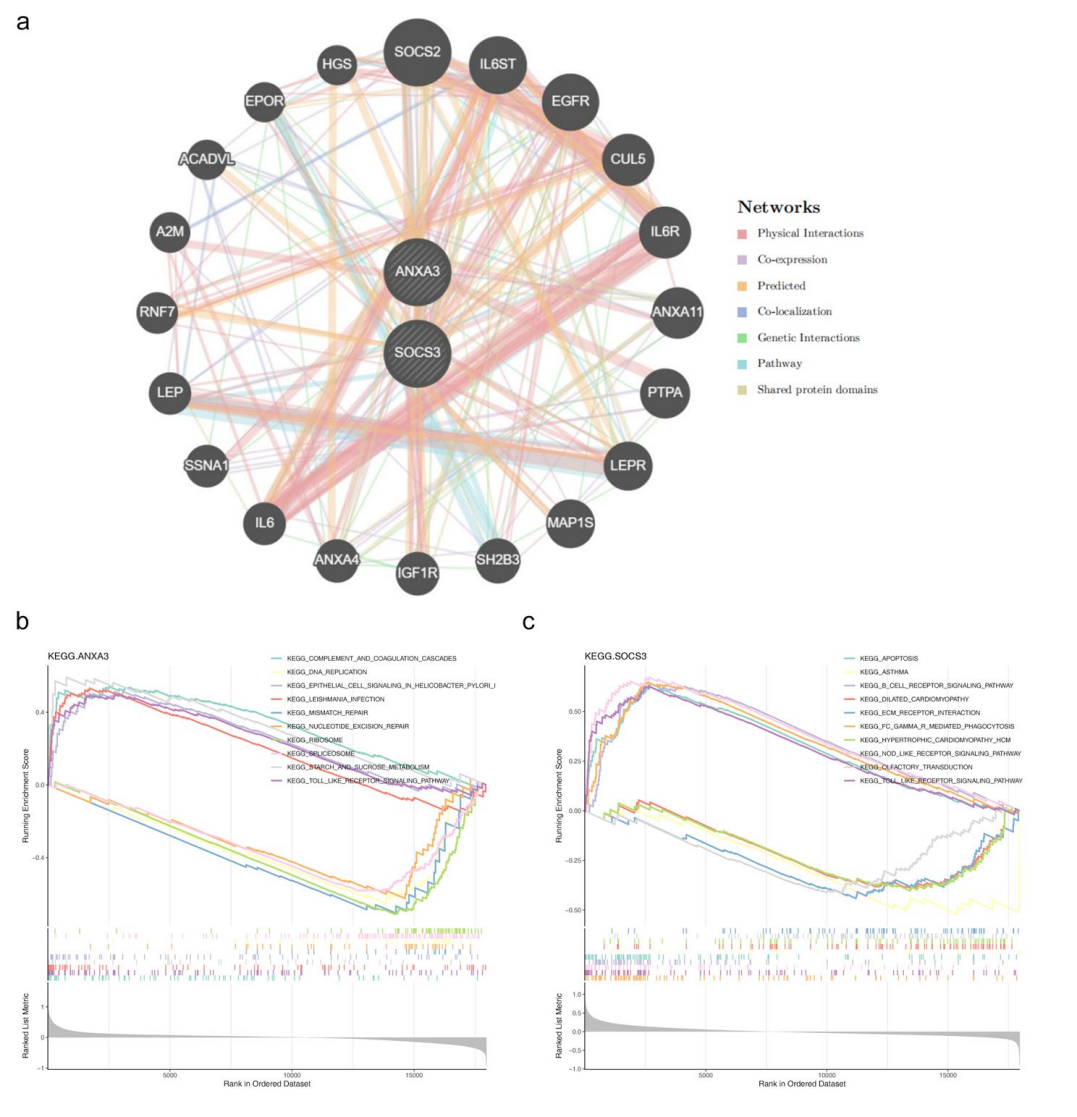
Pathway enrichment analysis of biomarkers (**a**) Network diagram showing genes associated with biomarkers in significant pathways. (**b**) Pathway enrichment analysis for *ANXA3* using GSEA. (**c**) Pathway enrichment analysis for *SOCS3*, illustrating differentially regulated pathways

Through prediction analysis, genes associated with the functions of *ANXA3* and *SOCS3* were identified, including LEP, IL6, IL6R, LEPR, RNF7, IL6ST, and SOCS2 (Fig. 5a). GSEA was performed to explore the signaling pathways enriched for these biomarkers. The top 10 paths with the maximum absolute NES values were chosen for visualization. *ANXA3* was positively correlated with pathways such as complement and coagulation cascades, epithelial cell signaling in Helicobacter pylori infection, Leishmania infection, starch and sucrose metabolism, and TLR signaling. Conversely, *ANXA3* showed significant negative correlations with DNA replication, mismatch repair, nucleotide excision repair, ribosome function, and spliceosome activity (Fig. 5b). In contrast, *SOCS3* was positively correlated with apoptosis, B cellular receptor signaling, phagocytosis mediated by Fc gamma receptor, NOD-like receptor and TLR pathways. *SOCS3* also exhibited negative correlations with asthma, dilated cardiomyopathy, ECM-receptor interaction, hypertrophic cardiomyopathy, and olfactory transduction (Fig. 5c).

### Biomarkers were closely linked to the immune microenvironment of AMI

**Fig. 6:**
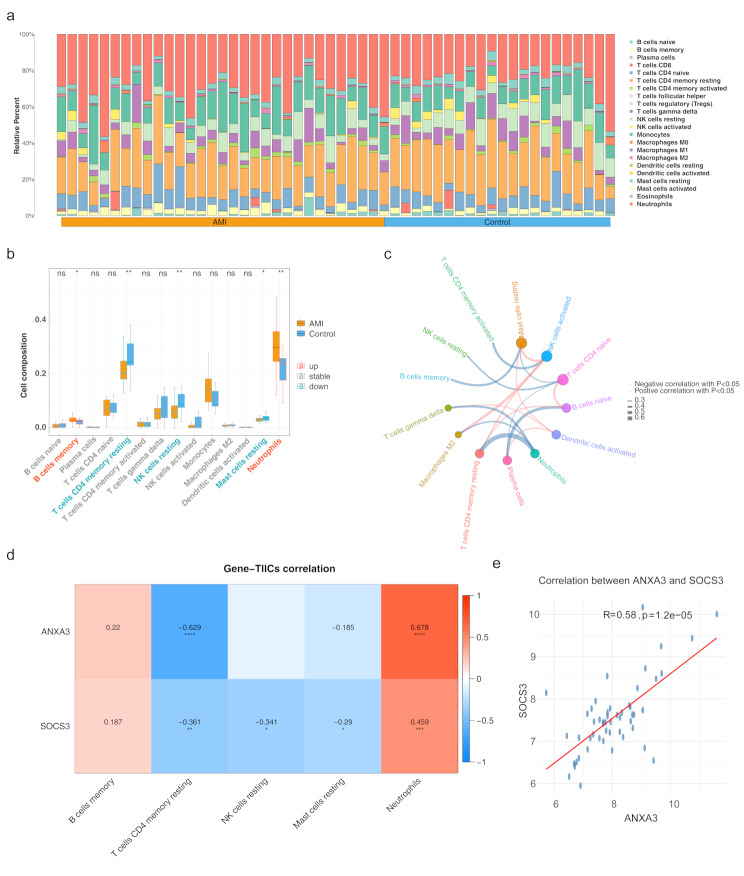
Immune infiltration in AMI (**a**) Overview of immune cell infiltration levels in AMI versus control samples. (**b**) Bar graph showing significant differences in specific immune cell types between AMI and control groups. (**c**) Correlation analysis among various immune cell types. (**d**) Correlation analysis between biomarkers and differentially infiltrated immune cell types, highlighting the immunological role in AMI. *p < 0.05, **p < 0.01 represented comparison of AMI with control group. (**e**) Correlation scatter plot

Immune infiltration analysis was conducted to investigate the relation between AMI and the immune micro-environment. In the GSE48060 dataset, the infiltration abundance of 22 immune cell types is shown in Fig. 6a. After excluding immune cells with an infiltration abundance of 0 in over 75% of the samples, 14 immune cell types were retained. Wilcoxon test results revealed significant differences in the infiltration levels of five immune cell types—memory B cells, resting memory CD4 T cells, resting NK cells, resting mast cells, and neutrophils—between the AMI and controls. Notably, memory B cells and neutrophils showed significantly higher infiltration in AMI compared to the control group (Fig. 6b). Correlation analysis indicated a significant positive association between activated NK cells and M2 macrophages (*p* < 0.05, cor = 0.48), as well as a significant negative correlation between resting memory CD4 T cells and neutrophils (*p* < 0.05, cor = -0.65) (Fig. 6c). Furthermore, *ANXA3* demonstrated the most significant positive correlation with neutrophils (*p* < 0.05, cor = 0.678) and the most significant negative correlation with resting memory CD4 T cells (*p* < 0.05, cor = -0.629) (Fig. 6d-e).

### Both *ANXA3* and *SOCS3* were regulated by YY1

**Fig. 7:**
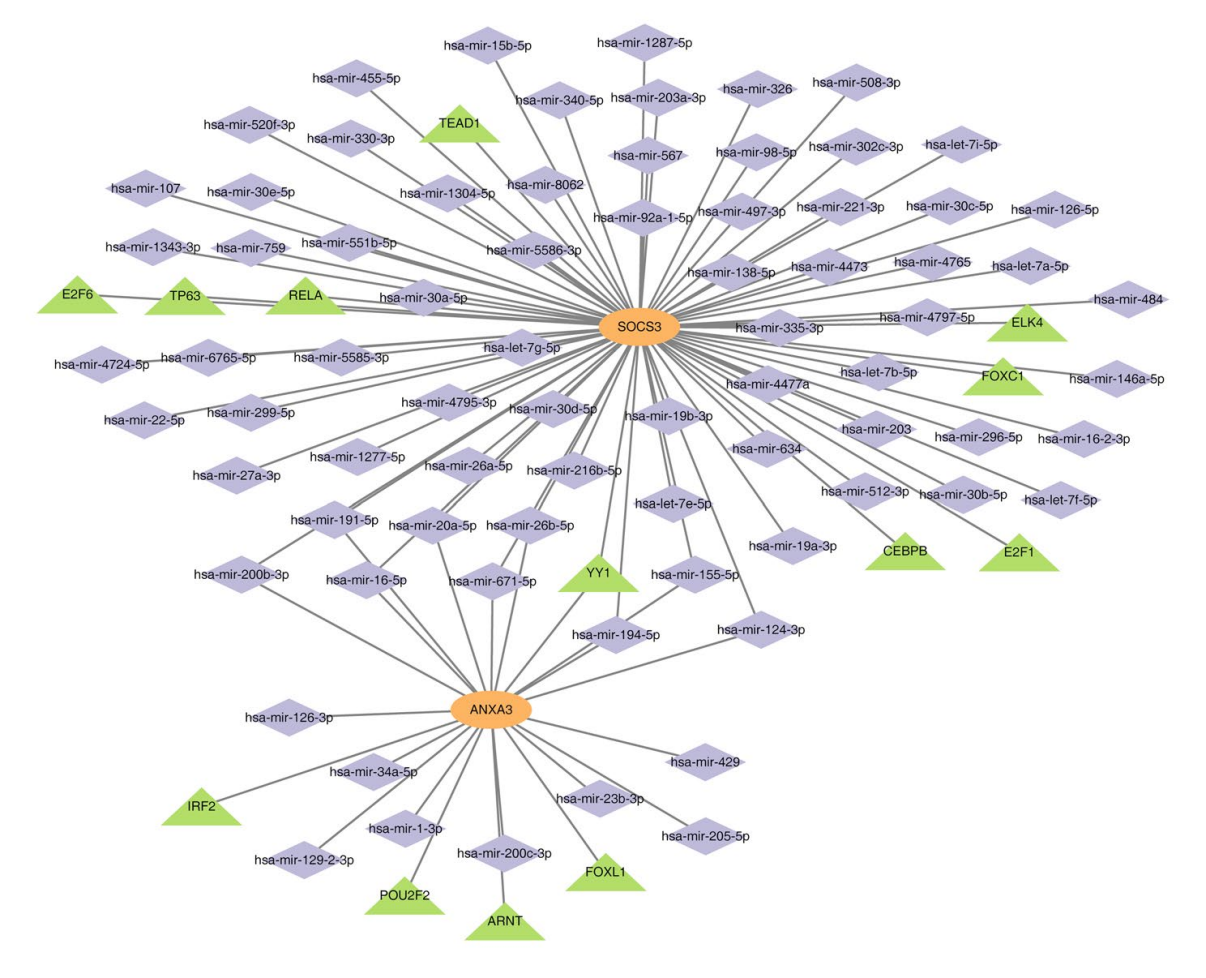
TF-miRNA-mRNA regulatory network, network illustrating interactions among transcription factors, miRNAs, and mRNA of biomarkers. This analysis highlights the regulatory impact of key transcription factors and miRNAs on *ANXA3* and *SOCS3*

Predictive analysis identified 76 miRNAs and 13 TFs targeting the biomarkers. A TF-miRNA-mRNA regulatory network was created, integrating the two biomarkers, 76 miRNAs, and 13 TFs (Fig. 7). Within this network, both *ANXA3* and *SOCS3* were concurrently regulated by the TF YY1 and several miRNAs, including hsa-miR-194-5p, hsa-miR-155-5p, hsa-miR-671-5p, hsa-miR-200b-3p, and hsa-miR-191-5p, among others.

### Affinity between biomarkers and their corresponding compounds

**Fig. 8:**
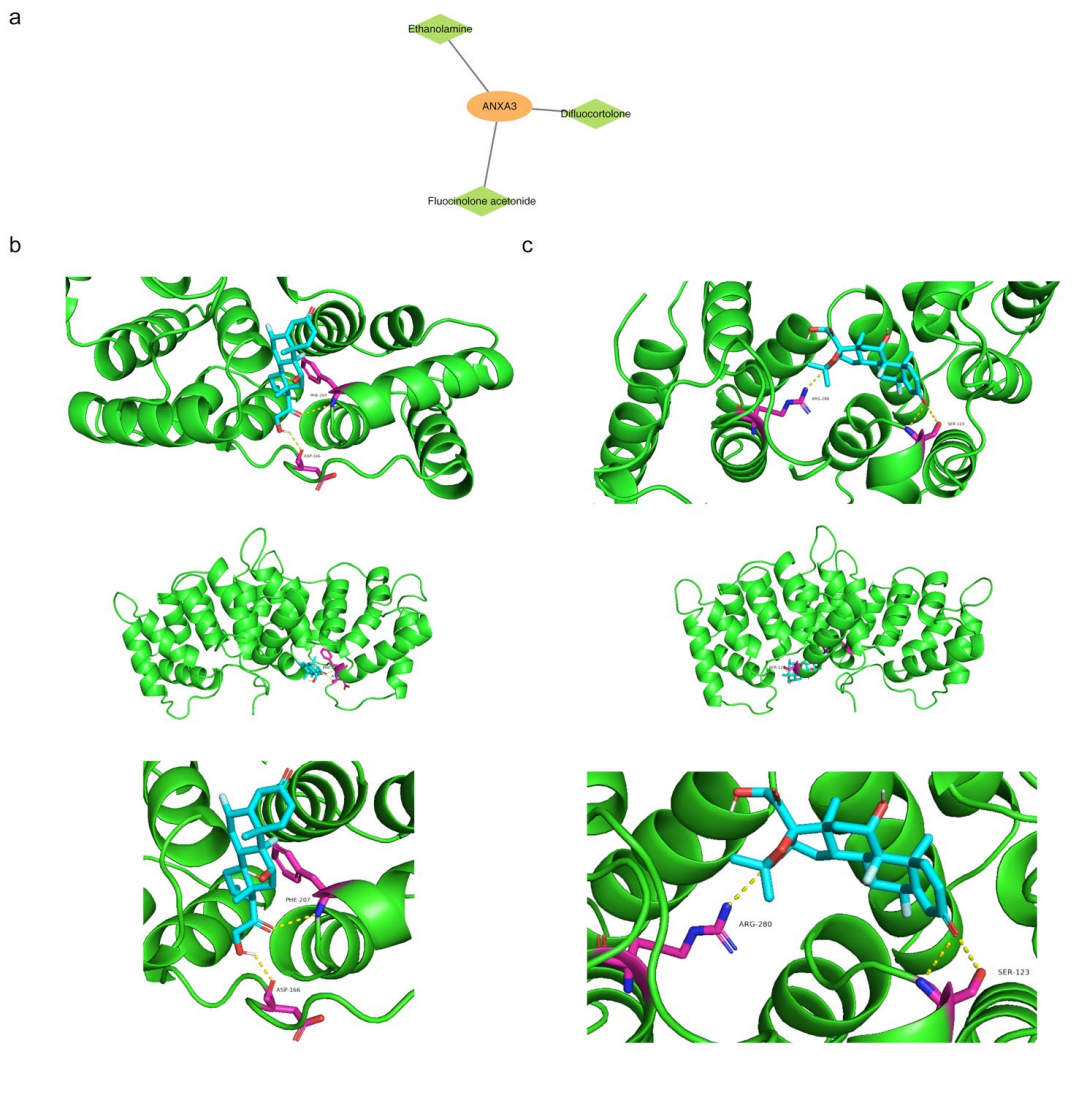
Drug target analysis and molecular docking (**a**) List of small molecule drugs targeting biomarkers, including their binding affinities. (**b**, **c**) Molecular docking results showing the interaction between *ANXA3* and targeted drugs, with detailed binding energy value

Drugs targeting *ANXA3* were identified, including ethanolamine, difluocortolone, and fluocinolone acetonide (Fig. 8a). Molecular docking studies aim to detect the interaction of these compounds and *ANXA3* (Table 2). Notably, *ANXA3* emerged covalent bonds the amino acid residues PHE-207 and ASP-166 of difluocortolone (binding energy = -8.4 kcal/mol) (Fig. 8b), as well as with ARG-280 and SER-123 of fluocinolone acetonide (binding energy = -8.7 kcal/mol) (Fig. 8c).

**Table 2:** Exceptional binding affinity of *ANXA3* with target compounds

Gene	Active Ingredient	Lowest Binding Energy (kcal/mol)
*ANXA3*	Difluocortolone	-8.4
*ANXA3*	Fluocinolone acetonide	-8.7

The RMSD between two protein structures, which quantifies differences in atomic positions, serves as an indicator of the stability of the overall system [35]. The results demonstrated that the RMSD for the *ANXA3*-diflucortolone complex stabilized after 8,000 picoseconds (ps) (Fig. 8d), with fluctuations around a constant value. At this point, the RMSD was approximately 1.3, indicating that the system had reached energy equilibrium. Similarly, the RMSD for the *ANXA3*-fluocinolone acetonide complex stabilized after 7,500 ps (Fig. 8e), with fluctuations around a constant value. The RMSD was approximately 1.8, further confirming that the system had achieved equilibrium.

### Six different cell types were annotated in the scRNA-seq

**Fig. 9:**
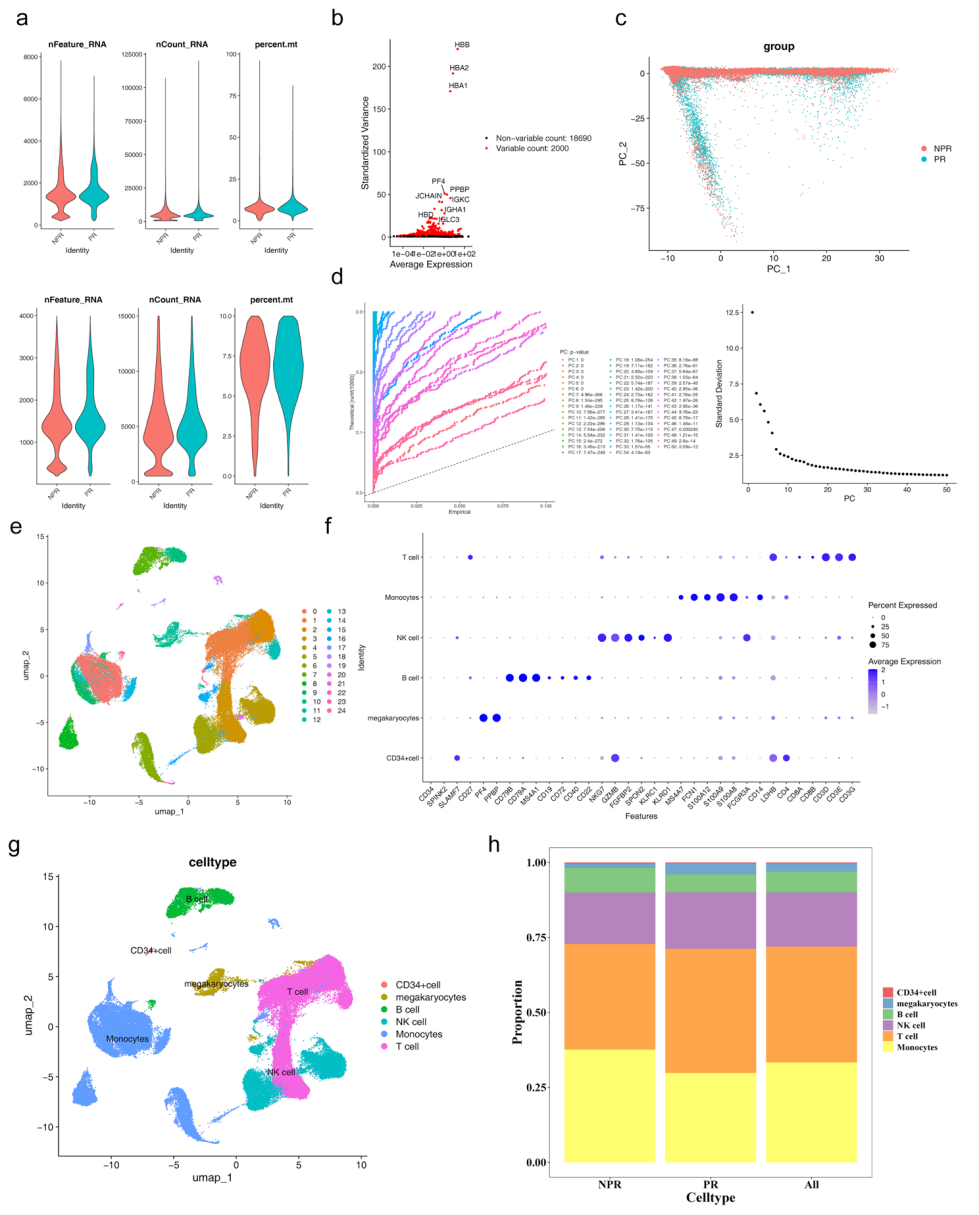
Single-cell RNA sequence analysis (**a**-1) Single-cell data analysis before quality control. (**a**-2) Single-cell data analysis after quality control. (**b**) Screening of high-resolution genes. (**c**) PCA results. (**d**) Elbow plot of PCA analysis. (**e**) UMAP clustering analysis results. (**f**) Expression of key genes across cell clusters. (**g**) Cellular annotation results. (**h**) Abundance of cell types across all samples

The scRNA-seq dataset in this study comprised samples from 5 patients with plaque rupture (PR) and 5 without plaque repture (NPR) individuals. Fig. 9a illustrates the distribution of gene counts, sequencing depth, and mitochondrial content ratios across all samples. After stringent quality control, 73,861 cells and 20,690 genes were retained for further analysis. Following data normalization, the top 2,000 most variable genes were identified, with the 10 most variable genes highlighted in Fig. 9b. PCA revealed that the data points for PR and NPR groups were closely clustered in principal component space, with no apparent batch effects (Fig. 9c). The elbow plot (Fig. 9d) indicated that the analysis stabilized after 30 principal components (PCs), the selection of the top 30 PCs for later analyses. UMAP clustering identified 25 distinct cell clusters (Fig. 9e), each characterized by multiple marker genes (Fig. 9f). Based on marker gene expression and annotations from reference literature, six cell types were identified across all samples, including CD34^+^ cells, megakaryocytes, B cells, NK cells, monocytes, and T cells (Fig. 9g). The relative abundance of these cell types across all samples is depicted in Fig. 9h.

### J. B cells and monocytes were defined as key cells, with differences in communication among the six types of cells between PR and NPR

**Fig. 10:**
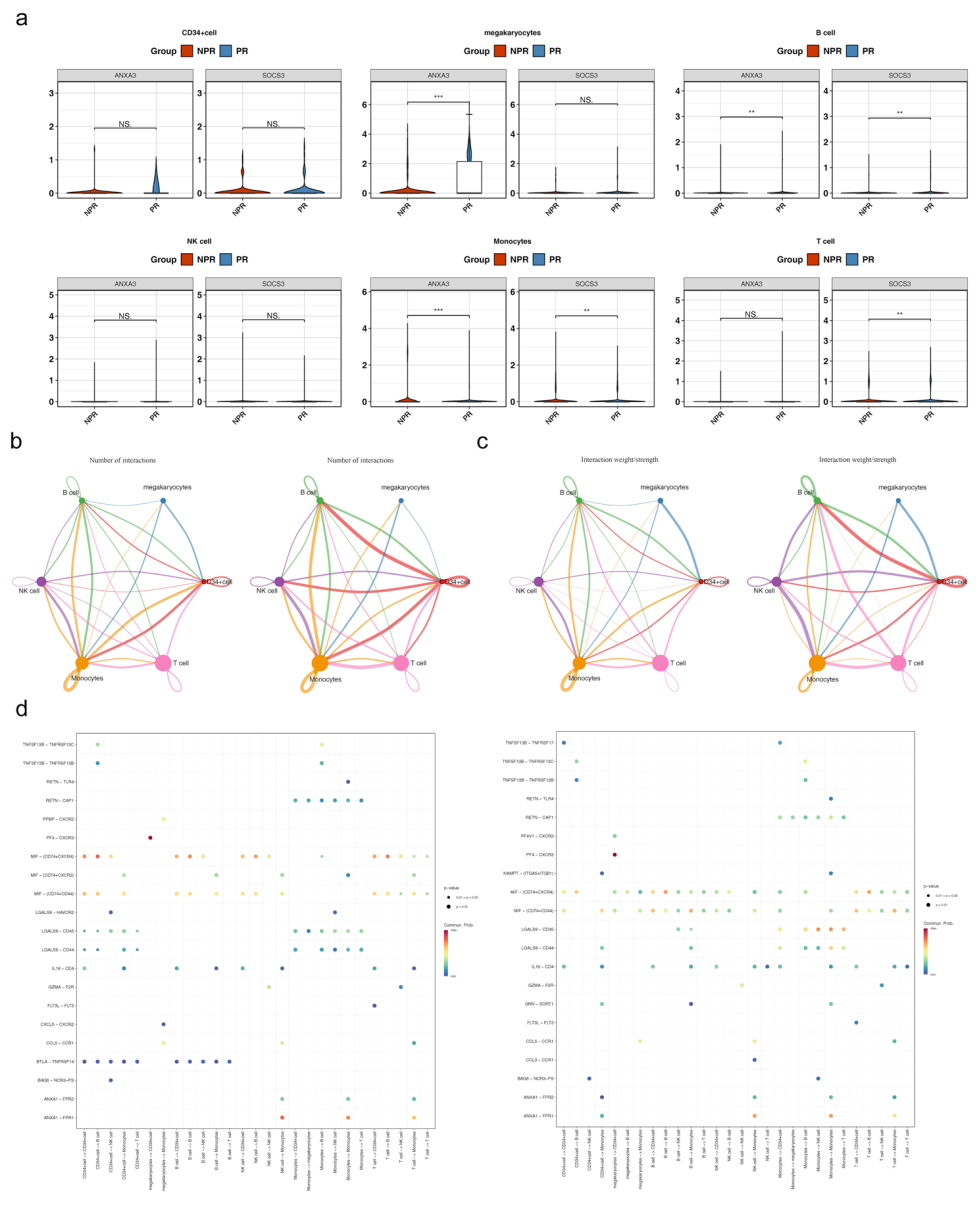
Cellular communication network analysis (**a**) Expression of biomarkers in key cell clusters. (**b**) Cellular communication network analysis in Plaque Rupture(PR) and without plaque rupture (NPR). (**c**) Cell connection weight network in PR and NPR. (**d**) Probability of ligand-receptor pair-regulated communication from specific cell populations to other cell groups. *p < 0.05, **p < 0.01, ***p < 0.001 represented comparison of PR with NPR group

To identify key cell clusters, the expression of *ANXA3* and *SOCS3* across different cell clusters was assessed. Significant differences in the expression of both biomarkers were observed in B cells and monocytes, thus designating these two cell types as key players (Fig. 10a). Dimensionality reduction and clustering analysis were performed separately on Monocytes and B cells, and they were re-clustered into different subgroups. B cells were clustered into 11 different subgroups, and Monocytes were clustered into 19 different subgroups (Supplementary material 6a-b). The marker gene expression levels of each cell subgroup in B cells and Monocytes are shown in Supplementary material 6c-d. The different subgroups of B cells and Monocytes were annotated, with the 19 clusters in B cells annotated as Transitional cells, Naïve cells, Double negative cells, Memory B cells, Regulatory plasma cells, and B1 cells, representing six cell subtypes. The 11 clusters in Monocytes were annotated as CD14 + CD16- monocytes, CD14-CD16 + monocytes, and CD14-CD16- monocytes, representing three cell subtypes (Supplementary material 6e-f). Cell communication network analysis revealed that NPR individuals exhibited more connections between monocytes and B cells, between B cells and CD34^+^ cells, compared to patients with PR. Conversely, patients with PR showed stronger associations between monocytes and CD34^+^ cells. Additionally, in patients with PR, B cells were connected to megakaryocytes, a connection not seen in NPR individuals. Conversely, CD34^+^ cells were linked to T cells in NPR individuals but not in patients with PR (Fig. 10b). The weighted cell connection network further highlighted that B cells in NPR individuals had stronger links with CD34^+^ cells, while in patients with PR, monocytes showed stronger links with CD34^+^ cells. Moreover, B cells were connected to megakaryocytes in patients with PR, and CD34^+^ cells were linked to T cells in NPR individuals, but these connections were absent in patients with PR (Fig. 10c). The probability of ligand-receptor pair-regulated communication between specific cell populations was illustrated in Fig. 10d. MIF^−^(CD74^+^CXCR4) and MIF^−^(CD74^+^CD44) were identified as potential receptors involved in extensive cell communication, with MIF^−^(CD74^+^CXCR4) being more likely to promote cell communication in the PR group.

Trajectory analysis of both B cells and monocytes showed similar patterns in PR and NPR groups, with biomarker expression peaking during monocyte stage 1 and remaining stable in B cells.

**Fig. 11:**
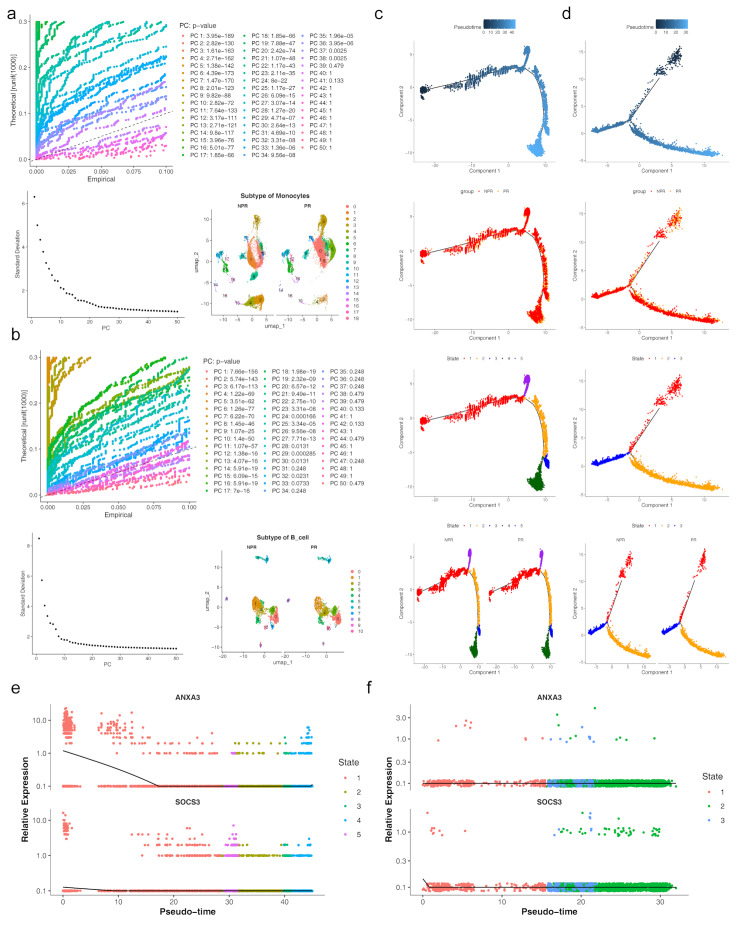
Monocyte and B cell analysis (**a**) Cluster analysis of monocytes. (**b**) Cluster analysis of B cells. (**c**) Monocyte cell trajectory analysis. (**d**) B cell trajectory analysis. (**e**) Expression of biomarkers at different stages of monocyte differentiation. (**f**) Expression of biomarkers at various stages of B cell differentiation

Initially, monocytes and B cells underwent separate dimensionality reduction and clustering. Monocytes were classified into 19 subpopulations (Fig. 11a), while B cells were grouped into 11 subpopulations (Fig. 11b). Cell trajectory analysis indicated that monocytes followed a trajectory with 1 root and 5 branches, gradually differentiating from left to right over time. The differentiation process was divided into 5 stages, with Stage 1 showing the longest developmental duration. Stage 4 represented the final differentiation stage, while Stage 5 had the shortest differentiation period, with high consistency between PR and NPR (Fig. 11c). B cells followed a trajectory with 1 root and 3 branches, with differentiation divided into 3 stages. Stage 1 had the longest duration, while Stage 3 was the shortest, and again, high consistency in differentiation was observed between the two groups (Fig. 11d). Expression analysis of biomarkers during monocyte differentiation revealed that *ANXA3* and *SOCS3* expression increased in Stage 1, followed by a gradual decrease in later stages (Fig. 11e). In contrast, during the differentiation of B cells, the expression levels of *ANXA3* and *SOCS3* did not exhibit any significant changes (Fig. 11f).

### Verification of biomarkers expression

**Fig. 12:**
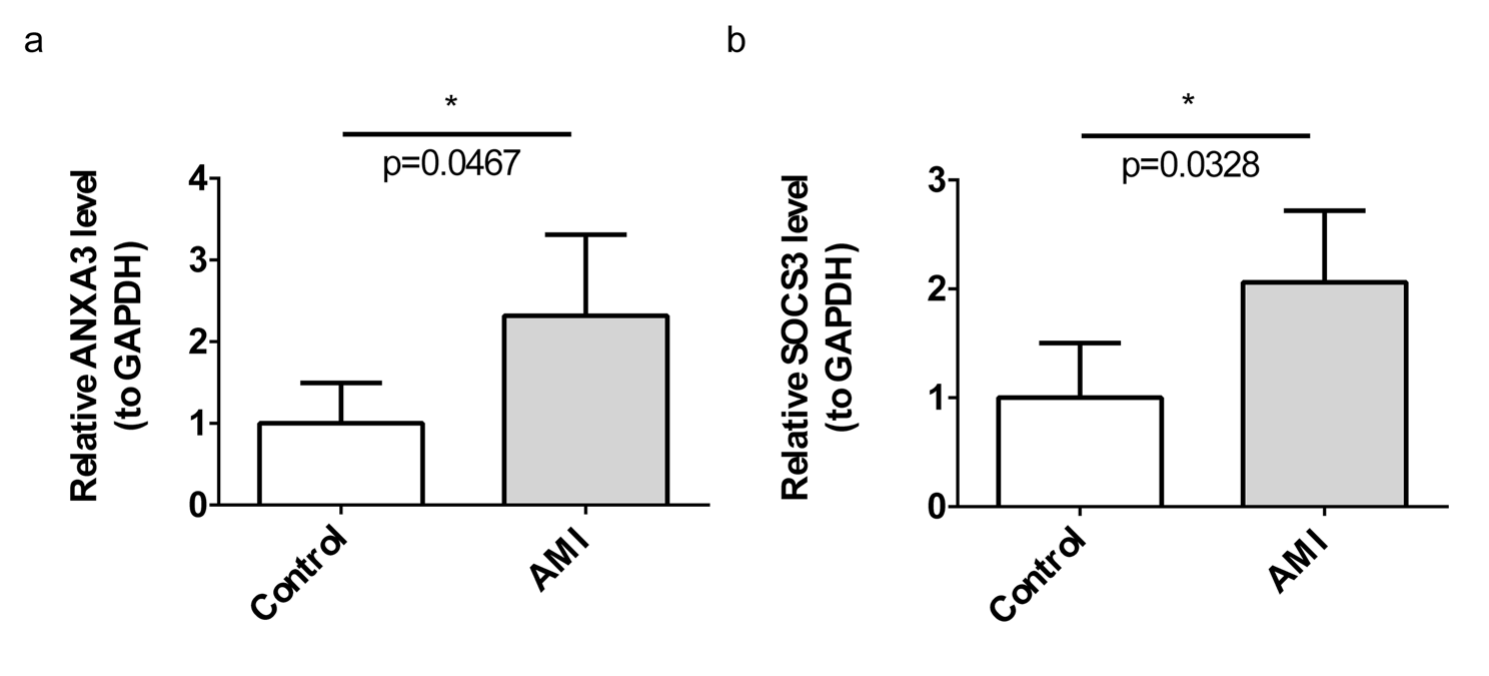
Validation of biomarker expression (**a**,** b**) RT-qPCR results validating the differential expression of *ANXA3* and *SOCS3* in AMI versus control samples, with statistical significance. *p < 0.05 represented comparison of AMI with control group

RT-qPCR results showed obvious difference in the expression levels of *ANXA3* and *SOCS3* between the AMI and control groups, with both biomarkers being significantly upregulated in the AMI group (*p* < 0.05) (Fig. 12a-b).

## Discussion

AMI represents a multifaceted cardiac condition involving intricate cellular and molecular pathways. Sphingolipids (SM) are integral to a variety of biological functions, including cell signaling, growth, and apoptosis [8, 9, 44], and are closely implicated in AMI pathogenesis, particularly in cellular stress response and inflammatory regulation [8, 9]. Sphingolipid metabolites, including ceramides and sphingomyelins, have been shown to modulate AMI progression by influencing cardiomyocyte survival through mechanisms of apoptosis and necrosis [45]. The regulation of cell signaling and inflammatory responses plays a pivotal role during AMI, directly affecting cardiomyocyte viability and potentially driving disease progression by modulating immune cell function and inflammatory cytokine release. Consequently, an in-depth investigation of sphingolipids offers promise for unraveling the underlying mechanisms of AMI and establishing a foundation for the development of novel therapeutic approaches.

*ANXA3* and *SOCS3*, as novel biomarkers for acute myocardial infarction (AMI), demonstrates significantly superior diagnostic performance compared to traditional markers CD34 and FLT3, with area under the ROC curve (AUC) exceeding 0.7, highlighting their stronger clinical relevance. Notably, these two biomarkers not only excel in AMI diagnosis but also show promising potential in diagnosing inflammatory responses [46] and lung cancer [47]. Unlike traditional biomarkers primarily involved in metabolic pathways, *ANXA3* and *SOCS3* are predominantly associated with immune-related biological pathways. This distinctive characteristic may underlie their diagnostic advantage, rendering them highly valuable for further research.

Annexin A3 (*ANXA3*) is a protein that participates in numerous pathophysiological processes, such as cell migration, proliferation and apoptosis, playing a particularly pivotal role in AMI. By modulating calcium signaling and inflammatory responses, *ANXA3* significantly contributes to cardiomyocyte survival and functional recovery [48]. Its elevated expression in other cardiovascular conditions [49] further supports its reliability as a biomarker for AMI. *SOCS3* functions as a critical negative modulator in immune and inflammatory responses via the JAK/STAT signaling pathway [50, 51]. In the context of AMI, *SOCS3* overexpression serves as a protective mechanism against myocardial injury and inflammation, aiming to contain inflammation and limit cellular damage. This regulatory role positions *SOCS3* as a potential therapeutic target, with its modulation offering promise for reducing myocardial damage post-AMI [52, 53]. *SOCS3* serves as a critical negative regulator of the JAK/STAT signaling pathway [49, 50], exerting inhibitory effects on immune and inflammatory responses. In the pathological context of acute myocardial infarction (AMI), its upregulated expression forms a protective defense mechanism by suppressing excessive inflammation and limiting cellular damage, thereby establishing its therapeutic potential [51, 52]. However, *SOCS3* demonstrates marked context-dependent functionality: while it modulates cardiomyocyte inflammation under IL-33 regulation during septic myocardial injury [54], it promotes trophoblast cell repair through B cells in recurrent miscarriage [55]. This functional versatility may originate from its participation in multiple signaling pathways beyond JAK/STAT inhibition (including PI3K/Akt and MAPK pathways [56]) and tissue microenvironment variations involving cytokine composition and cellular interactions [57]. These findings underscore the necessity for deeper investigation into *SOCS3*’s cell-specific regulatory mechanisms and the development of tailored therapeutic strategies addressing its pleiotropic roles. Additionally, *SOCS3* plays comparable roles in other cardiovascular diseases [58–60], reinforcing its potential as a universal biomarker across cardiovascular pathology. The consistent expression patterns of *ANXA3* and *SOCS3* in AMI and other cardiovascular diseases emphasize their universality and reliability. Moreover, the strong diagnostic performance of these biomarkers further validates their potential as effective targets for both diagnosing and treating AMI, providing a solid foundation for future clinical strategies and advancing research into their roles and mechanisms in cardiovascular disease.

GSEA in this study highlighted significant co-enrichment of *ANXA3* and *SOCS3* in the TLR pathway. The TLR pathway plays a pivotal role in triggering the activation of immune system. It empowers the body to identify pathogens and kick - start immune reactions. In AMI, TLR pathway activation may be critical for the myocardial injury response and repair process. Specifically, it prompts the discharge of pro-inflammatory cytokines like tumor gangrene factor-α and diverse interleukins. These cytokines function by attracting and activating immune cells, exacerbate myocardial tissue damage and inflammation [61]. Excessive activation of the TLR pathway is tightly connected to the pathogenesis of multiple cardiovascular diseases, influencing downstream signaling molecules like NF-κB, which not only modulate inflammatory responses but may also contribute to cardiomyocyte apoptosis and fibrosis [62, 63]. Thus, understanding the role of the TLR pathway in AMI can help to explore its molecular mechanisms and identifying potential therapeutic targets. Additionally, our analysis revealed a direct correlation between *ANXA3* and the complement and coagulation cascades pathway, suggesting its involvement in regulating thrombosis and inflammatory responses following MI.

Analysis of immune cell infiltration demonstrated that *ANXA3* has a positive connection with neutrophil infiltration. Conversely, it shows a negative correlation to the existence of resting memory CD4^+^ T cells. This indicates that *ANXA3* holds a crucial position in the regulation of immune cells. Neutrophils, which are the first responders during immune activation, rapidly infiltrate following MI, clearing necrotic tissue but potentially exacerbating inflammation [64]. *ANXA3* may modulate this process. Conversely, the diminished infiltration of memory CD4^+^ T cells indicates immune dysregulation in patients with AMI, which impairs cardiac repair [65]. Additionally, significant variations in the infiltration of dormant NK cells and mast cells were noted in individuals suffering from AMI. Both cell types are essential for regulating inflammation and promoting repair following cardiac injury. NK cells play a role in eliminating damaged myocardial tissue, while mast cells are involved in modulating inflammation. Research by Lin [66] and Zhu et al. [67] indicated that *SOCS3* negatively regulates immune responses through inhibition of the JAK/STAT signalling pathway. In the context of AMI, *SOCS3* has the potential to safeguard myocardial tissue by influencing the inflammatory response, thereby reinforcing its role as a possible biomarker for cardiovascular conditions and assisting in the development of innovative therapeutic approaches. In summary, both *ANXA3* and *SOCS3* appear to influence myocardial cell survival, apoptosis, and inflammation within the immune microenvironment, thereby impacting AMI progression and prognosis.

This study integrates TF, miRNA, and mRNA data to construct a regulatory network, elucidating the molecular mechanisms underlying AMI. Focus is placed on the roles of the transcription factor YY1 and miRNA hsa-miR-155-5p in regulating *ANXA3* and *SOCS3* expression. YY1, a multifunctional transcription factor, is critical in cellular processes such as differentiation, proliferation, and apoptosis, influencing both the physiological and pathological states of myocardial cells [68]. hsa-miR-155-5p is widely implicated in immune regulation, inflammation, and cardiovascular diseases, modulating key signalling pathways to control the inflammatory response and cardiac reconstruction after MI [69]. Within this regulatory network, YY1 and hsa-miR-155-5p collaboratively influence *ANXA3* and *SOCS3* expression. YY1 governs transcriptional regulation, while hsa-miR-155-5p affects post-translational regulation. This dual mechanism underscores the complexity of AMI’s molecular pathways and offers new perspectives for identifying potential therapeutic targets.

Single-cell analysis highlights B cells and monocytes as critical players in AMI pathology. Research has demonstrated that mature B lymphocytes influence cardiac recovery following MI in mice by secreting cytokines and regulating immune responses [70]. The absence of miR21 or Hif1a in marginal zone B cells improves cardiac function, suggesting that B cells participate in specific molecular mechanisms of cardiac repair [70]. Additionally, a reduction in the quantity of B regulatory cells (B-regs) in the circulation of patients with AMI is linked to the inflammatory response and immune imbalance that follows AMI [71]. The presence of B cells in the infarct zone correlates with infarct size and ventricular function, underscoring their role in AMI [72]. B cells also contribute to cardiac repair through antibody production and immune complex clearance [73–75]. Monocytes, as key innate immune cells, are critical for cardiac remodeling and heart failure progression following AMI [76]. A monocyte/macrophage transition map of the ischemic heart, developed by Giuseppe Rizzo’s team, provides valuable insights for advancing heart repair after ischemic injury [77]. Additionally, research by Zheng Dong suggests that MI induces monocytes to acquire trained immunity, accelerating atherosclerosis [78]. Monocytes are also crucial for cardiac injury and the repair process during AMI. They achieve this by releasing cytokines and regulating inflammatory reactions [39, 79, 80]. Furthermore, pseudotime trajectory analysis revealed high differentiation consistency between the two single-cell populations, though with distinct annotated cell subtypes. This finding further supports the diagnostic potential of monocytes in myocardial infarction [81], while B cells appear to influence disease progression through signal modulation by other immune factors [82].

The interactions between immune cells further influence AMI progression. Specifically, the interplay between B cells and monocytes regulates the immune microenvironment and supports cardiac repair [83–85]. In this study, significant changes in *ANXA3* expression were observed during monocyte differentiation, suggesting its involvement in immune responses and repair following AMI. In contrast, *SOCS3* expression remained largely unchanged, warranting additional investigation. It is hypothesized that the altered expression of *ANXA3* is linked to immune regulation and cardiac repair processes, positioning it as a possible therapeutic target. Future research should delve into the precise mechanisms by which *ANXA3* contributes to AMI, as well as explore the functional significance of *SOCS3*.

While this study demonstrates promising findings, several limitations should be acknowledged. The identification and validation of biomarkers primarily relied on public databases, necessitating further verification in larger independent cohorts. Although bioinformatics analysis aligned with RT-qPCR results, the validation sample size was limited and lacked functional experiments. Subsequent studies should expand sample sizes and employ *ANXA3*/*SOCS3* knockout or overexpression models to establish their causal relationship with the TLR pathway. Additionally, the GEO datasets used lacked essential markers for standard AML classification (including morphological, immunophenotypic, and molecular genetic markers), restricting precise subtype analysis. For immune infiltration analysis, peripheral blood data showed suboptimal compatibility with the CIBERSORT algorithm; alternative algorithms such as quanTIseq or EPIC are recommended for optimization.Future research will focus on validating the clinical utility of these biomarkers, elucidating their mechanistic roles in AMI pathogenesis, and exploring their therapeutic potential.

## Conclusion

This study comprehensively investigates the essential roles of *ANXA3* and *SOCS3* as indicators for AMI, focusing specifically on their association with SM. It offers a detailed understanding of how these biomarkers influence immune responses and the molecular processes driving AMI, especially their regulation of Toll-like receptor signaling pathways. The application of ANN technology further demonstrates the significant potential of advanced computational approaches in enhancing the diagnostic precision of complex diseases like AMI. These findings suggest that targeting these biomarkers could substantially improve clinical outcomes in AMI management. By incorporating these novel biomarkers, the study not only illuminates the disease’s underlying mechanisms but also identifies potential therapeutic targets, enhances predictions of disease progression, and paves the way for innovative advancements in AMI treatment.

## Electronic Supplementary Material

Below is the link to the electronic supplementary material.

Supplementary Material 1

Supplementary Material 2

Supplementary Material 3

Supplementary Material 4

Supplementary Material 5

Supplementary Material 6

## Data Availability

The datasets (GSE48060,GSE123342,GSE269269) analysed during the current study are available in the Gene Expression Omnibus (GEO) repository, (https://www.ncbi.nlm.nih.gov/gds).

## Abbreviation

AMI: Acute Myocardial Infarction
SM: Sphingolipid Metabolism
SMRGs: Sphingolipid Metabolism-Related Genes
DEGs: Differentially Expressed Genes
PPI: Protein-Protein Interaction
ROC: Receiver Operating Characteristic
ANN: Artificial Neural Network
scRNA-seq: Single-cell RNA sequencing
RT-qPCR: Reverse Transcription Quantitative Polymerase Chain Reaction
WGCNA: Weighted Gene Co-expression Network Analysis
SVM-RFE: Support Vector Machine Recursive Feature Elimination
XGBoost: EXtreme Gradient Boosting
GO: Gene Ontology
KEGG: Kyoto Encyclopedia of Genes and Genomes
BP: Biological Process
MF: Molecular Function
CC: Cellular Component
GSEA: Gene Set Enrichment Analysis
AUC: Area Under the Curve
TLR: Toll-like Receptor
TF: Transcription Factor
miRNA: MicroRNA
mRNA: Messenger RNA
PCA: Principal Component Analysis
UMAP: Uniform Manifold Approximation and Projection
NK: Natural Killer
T cells: T lymphocytes
B cells: B lymphocytes
PR: Plaque Rupture
NPR: Non-Plaque Rupture
RMSD: Root-Mean-Square Deviation
MD: Molecular Dynamics
JAK/STAT: Janus Kinase/Signal Transducer and Activator of Transcription
NF-κB: Nuclear Factor kappa-light-chain-enhancer of activated B cells
MIF: Macrophage Migration Inhibitory Factor
CD74: Cluster of Differentiation 74
CXCR4: C-X-C Chemokine Receptor Type 4
CD44: Cluster of Differentiation 44

## References

[1] Reed GW, Rossi JE, Cannon CP. Acute myocardial infarction. Lancet. 2017;389:197–210. 27502078 10.1016/S0140-6736(16)30677-8

[2] Gupta S, Singh KN, Bapat V, Mishra V, Agarwal DK, Gupta P. Diagnosis of acute myocardial infarction: CK-MB versus cTn-T in Indian patients. Indian J Clin Biochem. 2008;23:89–91. 23105729 10.1007/s12291-008-0021-7 PMC3453645

[3] Bacci M, Lorito N, Smiriglia A, Morandi A. Fat and furious: lipid metabolism in antitumoral therapy response and resistance. Trends Cancer. 2021;7:198–213. 33281098 10.1016/j.trecan.2020.10.004

[4] Alessenko AV. The role of sphingomyelin cycle metabolites in transduction of signals of cell proliferation, differentiation and death. Membr Cell Biol. 2000;13:303–20. 10779176

[5] Olivera A, Buckley NE, Spiegel S. Sphingomyelinase and cell-permeable ceramide analogs stimulate cellular proliferation in quiescent Swiss 3T3 fibroblasts. J Biol Chem. 1992;267:26121–7. 1464623

[6] Moles A, Tarrats N, Morales A, Domínguez M, Bataller R, Caballería J, et al. Acidic sphingomyelinase controls hepatic stellate cell activation and in vivo liver fibrogenesis. Am J Pathol. 2010;177:1214–24. 20651240 10.2353/ajpath.2010.091257 PMC2928955

[7] Hadas Y, Vincek AS, Youssef E, Żak MM, Chepurko E, Sultana N, et al. Altering sphingolipid metabolism attenuates cell death and inflammatory response after myocardial infarction. Circulation. 2020;141:916–30. 31992066 10.1161/CIRCULATIONAHA.119.041882 PMC7135928

[8] Egom EE, Mamas MA, Clark AL. The potential role of sphingolipid-mediated cell signaling in the interaction between hyperglycemia, acute myocardial infarction and heart failure. Expert Opin Ther Targets. 2012;16:791–800. 22762510 10.1517/14728222.2012.699043

[9] Zhang F, Xia Y, Yan W, Zhang H, Zhou F, Zhao S, et al. Sphingosine 1-phosphate signaling contributes to cardiac inflammation, dysfunction, and remodeling following myocardial infarction. Am J Physiol Heart Circ Physiol. 2016;310:H250–61. 26589326 10.1152/ajpheart.00372.2015

[10] Jovic D, Liang X, Zeng H, Lin L, Xu F, Luo Y. Single-cell RNA sequencing technologies and applications: a brief overview. Clin Transl Med. 2022;12:e694. 35352511 10.1002/ctm2.694 PMC8964935

[11] Li S, Ge T, Xu X, Xie L, Song S, Li R, et al. Integrating scRNA-seq to explore novel macrophage infiltration-associated biomarkers for diagnosis of heart failure. BMC Cardiovasc Disord. 2023;23:560. 37974098 10.1186/s12872-023-03593-1 PMC10652463

[12] Wang K, Zheng Q, Liu X, Geng B, Dong N, Shi J. Identifying hub genes of calcific aortic valve disease and revealing the immune infiltration landscape based on multiple WGCNA and single-cell sequence analysis. Front Immunol. 2022;13:1035285. 36405745 10.3389/fimmu.2022.1035285 PMC9673246

[13] Zhong C, Si Y, Yang H, Zhou C, Chen Y, Wang C, et al. Identification of monocyte-associated pathways participated in the pathogenesis of pulmonary arterial hypertension based on omics-data. Pulm Circ. 2023;13:e12319. 38130888 10.1002/pul2.12319 PMC10733707

[14] Heiser CN, Simmons AJ, Revetta F, McKinley ET, Ramirez-Solano MA, Wang J, et al. Molecular cartography uncovers evolutionary and microenvironmental dynamics in sporadic colorectal tumors. Cell. 2023;186:5620–e3716. 38065082 10.1016/j.cell.2023.11.006 PMC10756562

[15] Hu W, Zeng H, Shi Y, Zhou C, Huang J, Jia L, et al. Single-cell transcriptome and translatome dual-omics reveals potential mechanisms of human oocyte maturation. Nat Commun. 2022;13:5114. 36042231 10.1038/s41467-022-32791-2 PMC9427852

[16] Macosko EZ, Basu A, Satija R, Nemesh J, Shekhar K, Goldman M, et al. Highly parallel genome-wide expression profiling of individual cells using nanoliter droplets. Cell. 2015;161:1202–14. 26000488 10.1016/j.cell.2015.05.002 PMC4481139

[17] Yu T, Zhang C, Song W, Zhao X, Cheng Y, Liu J, et al. Single-cell RNA-seq and single-cell bisulfite-sequencing reveal insights into Yak preimplantation embryogenesis. J Biol Chem. 2024;300:105562. 38097189 10.1016/j.jbc.2023.105562 PMC10821408

[18] Chi H, Peng G, Yang J, Zhang J, Song G, Xie X, et al. Machine learning to construct sphingolipid metabolism genes signature to characterize the immune landscape and prognosis of patients with uveal melanoma. Front Endocrinol (Lausanne). 2022;13:1056310. 36568076 10.3389/fendo.2022.1056310 PMC9772281

[19] Ritchie ME, Phipson B, Wu D, Hu Y, Law CW, Shi W, et al. Limma powers differential expression analyses for RNA-sequencing and microarray studies. Nucleic Acids Res. 2015;43:e47. 25605792 10.1093/nar/gkv007 PMC4402510

[20] Gustavsson EK, Zhang D, Reynolds RH, Garcia-Ruiz S, Ryten M. Ggtranscript: an R package for the visualization and interpretation of transcript isoforms using ggplot2. Bioinformatics. 2022;38:3844–6. 35751589 10.1093/bioinformatics/btac409 PMC9344834

[21] Gu Z, Hübschmann D. Make interactive complex heatmaps in R. Bioinformatics. 2022;38:1460–2. 34864868 10.1093/bioinformatics/btab806 PMC8826183

[22] Hänzelmann S, Castelo R, Guinney J. GSVA: gene set variation analysis for microarray and RNA-seq data. BMC Bioinformatics. 2013;14:7. 23323831 10.1186/1471-2105-14-7 PMC3618321

[23] Langfelder P, Horvath S. WGCNA: an R package for weighted correlation network analysis. BMC Bioinformatics. 2008;9:559. 19114008 10.1186/1471-2105-9-559 PMC2631488

[24] Tomás CC, Oliveira E, Sousa D, Uba-Chupel M, Furtado G, Rocha C, et al. Proceedings of the 3rd IPLeiria’s international health congress: Leiria, Portugal. 6–7 May 2016. BMC Health Serv Res. 2016;16 Suppl 3:200. 10.1186/s12913-016-1423-5 PMC4943498 27409075

[25] Jia A, Xu L, Wang Y. Venn diagrams in bioinformatics. Brief Bioinform. 2021;22. 10.1093/bib/bbab108 33839742

[26] Yu G, Wang LG, Han Y, He QY. ClusterProfiler: an R package for comparing biological themes among gene clusters. Omics. 2012;16:284–7. 22455463 10.1089/omi.2011.0118 PMC3339379

[27] Wang L, Wang D, Yang L, Zeng X, Zhang Q, Liu G, et al. Cuproptosis related genes associated with Jab1 shapes tumor microenvironment and pharmacological profile in nasopharyngeal carcinoma. Front Immunol. 2022;13:989286. 36618352 10.3389/fimmu.2022.989286 PMC9816571

[28] Zhang Z, Zhao Y, Canes A, Steinberg D, Lyashevska O. Predictive analytics with gradient boosting in clinical medicine. Ann Transl Med. 2019;7:152. 31157273 10.21037/atm.2019.03.29 PMC6511546

[29] Hou N, Li M, He L, Xie B, Wang L, Zhang R, et al. Predicting 30-days mortality for MIMIC-III patients with sepsis-3: a machine learning approach using XGboost. J Transl Med. 2020;18:462. 33287854 10.1186/s12967-020-02620-5 PMC7720497

[30] Robin X, Turck N, Hainard A, Tiberti N, Lisacek F, Sanchez JC, et al. pROC: an open-source package for R and S + to analyze and compare ROC curves. BMC Bioinformatics. 2011;12:77. 21414208 10.1186/1471-2105-12-77 PMC3068975

[31] Newman AM, Liu CL, Green MR, Gentles AJ, Feng W, Xu Y, et al. Robust enumeration of cell subsets from tissue expression profiles. Nat Methods. 2015;12:453–7. 25822800 10.1038/nmeth.3337 PMC4739640

[32] Shannon P, Markiel A, Ozier O, Baliga NS, Wang JT, Ramage D, et al. Cytoscape: a software environment for integrated models of biomolecular interaction networks. Genome Res. 2003;13:2498–504. 14597658 10.1101/gr.1239303 PMC403769

[33] Saikia S, Bordoloi M. Molecular docking: challenges, advances and its use in drug discovery perspective. Curr Drug Targets. 2019;20:501–21. 30360733 10.2174/1389450119666181022153016

[34] Guterres H, Im W. CHARMM-GUI-Based induced fit docking workflow to generate reliable protein-ligand binding modes. J Chem Inf Model. 2023;63:4772–9. 37462607 10.1021/acs.jcim.3c00416 PMC10428204

[35] Rai H, Barik A, Singh YP, Suresh A, Singh L, Singh G, et al. Molecular docking, binding mode analysis, molecular dynamics, and prediction of admet/toxicity properties of selective potential antiviral agents against SARS-CoV-2 main protease: an effort toward drug repurposing to combat COVID-19. Mol Divers. 2021;25:1905–27. 33582935 10.1007/s11030-021-10188-5 PMC7882058

[36] Selvaraj G, Kaliamurthi S, Peslherbe GH, Wei DQ. Identifying potential drug targets and candidate drugs for COVID-19: biological networks and structural modeling approaches. F1000Res. 2021;10:127. 33968364 10.12688/f1000research.50850.1 PMC8080978

[37] Sharma P, Sharma P, Ahmad S, Kumar A. Chikungunya virus vaccine development: through computational proteome exploration for finding of HLA and cTAP binding novel epitopes as vaccine candidates. Int J Pept Res Ther. 2022;28:50. 35069056 10.1007/s10989-021-10347-0 PMC8762984

[38] Hao Y, Hao S, Andersen-Nissen E, Mauck WM 3rd, Zheng S, Butler A, et al. Integrated analysis of multimodal single-cell data. Cell. 2021;184:3573–e8729. 10.1016/j.cell.2021.04.048 PMC8238499 34062119

[39] Qian J, Gao Y, Lai Y, Ye Z, Yao Y, Ding K, et al. Single-Cell RNA sequencing of peripheral blood mononuclear cells from acute myocardial infarction. Front Immunol. 2022;13:908815. 35844519 10.3389/fimmu.2022.908815 PMC9278132

[40] Sanz I, Wei C, Jenks SA, Cashman KS, Tipton C, Woodruff MC, et al. Challenges and opportunities for consistent classification of human B cell and plasma cell populations. Front Immunol. 2019;10:2458. 31681331 10.3389/fimmu.2019.02458 PMC6813733

[41] Jin S, Guerrero-Juarez CF, Zhang L, Chang I, Ramos R, Kuan CH, et al. Inference and analysis of cell-cell communication using cellchat. Nat Commun. 2021;12:1088. 33597522 10.1038/s41467-021-21246-9 PMC7889871

[42] Trapnell C, Cacchiarelli D, Grimsby J, Pokharel P, Li S, Morse M, et al. The dynamics and regulators of cell fate decisions are revealed by pseudotemporal ordering of single cells. Nat Biotechnol. 2014;32:381–6. 24658644 10.1038/nbt.2859 PMC4122333

[43] Livak KJ, Schmittgen TD. Analysis of relative gene expression data using real-time quantitative PCR and the 2(-Delta delta C(T)) method. Methods. 2001;25:402–8. 11846609 10.1006/meth.2001.1262

[44] Pavoine C, Pecker F. Sphingomyelinases: their regulation and roles in cardiovascular pathophysiology. Cardiovasc Res. 2009;82:175–83. 19176603 10.1093/cvr/cvp030 PMC2855341

[45] Kawabori M, Kacimi R, Karliner JS, Yenari MA. Sphingolipids in cardiovascular and cerebrovascular systems: pathological implications and potential therapeutic targets. World J Cardiol. 2013;5:75–86. 23675553 10.4330/wjc.v5.i4.75 PMC3653015

[46] Hosseini L, Soltani-Zangbar MS, Abolhasanpour N, Hosseini M, Delkhosh A, Dolati S, et al. The effect of anti-CD20 on inflammation and histopathological alternations in rat photothrombotic ischemic stroke model. Immunol Res. 2025;73:75. 40266449 10.1007/s12026-025-09630-9

[47] Tan Q, Gao D, Hu X. FOXD1-activated ANXA3 facilitates cisplatin resistance of lung cancer cells via promoting ANXA4 expression. Naunyn Schmiedebergs Arch Pharmacol. 2025. 10.1007/s00210-025-04005-1. 10.1007/s00210-025-04005-1 40095055

[48] Meng H, Zhang Y, An ST, Chen Y. Annexin A3 gene silencing promotes myocardial cell repair through activation of the PI3K/Akt signaling pathway in rats with acute myocardial infarction. J Cell Physiol. 2019;234:10535–46. 30456911 10.1002/jcp.27717

[49] Huang K, Fan X, Jiang Y, Jin S, Huang J, Pang L, et al. Integrative identification of hub genes in development of atrial fibrillation related stroke. PLoS ONE. 2023;18:e0283617. 36952494 10.1371/journal.pone.0283617 PMC10035830

[50] Xiao C, Hong H, Yu H, Yuan J, Guo C, Cao H, et al. MiR-340 affects gastric cancer cell proliferation, cycle, and apoptosis through regulating SOCS3/JAK-STAT signaling pathway. Immunopharmacol Immunotoxicol. 2018;40:278–83. 29658372 10.1080/08923973.2018.1455208

[51] Zhou C, Huang J, Chen J, Lai J, Zhu F, Xu X, et al. CYP2J2-Derived EETs attenuated angiotensin II-induced adventitial remodeling via reduced inflammatory response. Cell Physiol Biochem. 2016;39:721–39. 27459385 10.1159/000445663

[52] Meng H, Wang X, Ruan J, Chen W, Meng F, Yang P. High expression levels of the SOCS3 gene are associated with acute myocardial infarction. Genet Test Mol Biomarkers. 2020;24:443–50. 32589469 10.1089/gtmb.2020.0040

[53] Nagata T, Yasukawa H, Kyogoku S, Oba T, Takahashi J, Nohara S, et al. Cardiac-specific SOCS3 deletion prevents in vivo myocardial ischemia reperfusion injury through sustained activation of cardioprotective signaling molecules. PLoS ONE. 2015;10:e0127942. 26010537 10.1371/journal.pone.0127942 PMC4444323

[54] Weng D, Shi W, Hu Y, Su Y, Li A, Wei S, et al. Neutralization of IL-33 ameliorates septic myocardial injury through anti-inflammatory, anti-oxidative, and anti-apoptotic by regulating the NF-κB/STAT3/SOCS3 signaling pathway. Biochem Pharmacol. 2025;237:116954. 40258576 10.1016/j.bcp.2025.116954

[55] Cao H, Li R, Ling L, Ni G. PF4 silencing promotes trophoblast cell proliferation, migration, invasion and EMT by regulating SOCS3/STAT3 signaling pathway. Endocr Metab Immune Disord Drug Targets; 2024. 10.2174/0118715303299470240723060939 PMC13284655 39694960

[56] Wang S, Du Q, Sun J, Geng S, Zhang Y. Investigation of the mechanism of isobavachalcone in treating rheumatoid arthritis through a combination strategy of network pharmacology and experimental verification. J Ethnopharmacol. 2022;294:115342. 35525528 10.1016/j.jep.2022.115342

[57] Nakayama Y, Igarashi K, Jin Z, Yamaguchi A, Ganss B, Ogata Y. Conditioned medium from cultured cementoblasts upregulates amelotin gene expression via the SOCS3 signaling pathway. J Periodontal Implant Sci. 2025. 10.5051/jpis.2403080154. 10.5051/jpis.2403080154 PMC12411138 40047181

[58] Li Y, Kinzenbaw DA, Modrick ML, Pewe LL, Faraci FM. Context-dependent effects of SOCS3 in angiotensin II-induced vascular dysfunction and hypertension in mice: mechanisms and role of bone marrow-derived cells. Am J Physiol Heart Circ Physiol. 2016;311:H146–56. 27106041 10.1152/ajpheart.00204.2016 PMC4967211

[59] Recio C, Oguiza A, Mallavia B, Lazaro I, Ortiz-Muñoz G, Lopez-Franco O, et al. Gene delivery of suppressors of cytokine signaling (SOCS) inhibits inflammation and atherosclerosis development in mice. Basic Res Cardiol. 2015;110:8. 25604439 10.1007/s00395-014-0458-1

[60] Unsöld B, Bremen E, Didié M, Hasenfuss G, Schäfer K. Differential PI3K signal transduction in obesity-associated cardiac hypertrophy and response to ischemia. Obes (Silver Spring). 2015;23:90–9. 10.1002/oby.20888 25175008

[61] Lipps C, Nguyen JH, Pyttel L, Lynch TLt, Liebetrau C, Aleshcheva G, et al. N-terminal fragment of cardiac myosin binding protein-C triggers pro-inflammatory responses in vitro. J Mol Cell Cardiol. 2016;99:47–56. 27616755 10.1016/j.yjmcc.2016.09.003 PMC5107329

[62] Fernández-Velasco M, Prieto P, Terrón V, Benito G, Flores JM, Delgado C, et al. NOD1 activation induces cardiac dysfunction and modulates cardiac fibrosis and cardiomyocyte apoptosis. PLoS ONE. 2012;7:e45260. 23028889 10.1371/journal.pone.0045260 PMC3445482

[63] Yoshida K, Abe K, Ishikawa M, Saku K, Shinoda-Sakamoto M, Ishikawa T, et al. Inhibition of TLR9-NF-κB-mediated sterile inflammation improves pressure overload-induced right ventricular dysfunction in rats. Cardiovasc Res. 2019;115:658–68. 30239623 10.1093/cvr/cvy209

[64] Daseke MJ 2nd, Chalise U, Becirovic-Agic M, Salomon JD, Cook LM, Case AJ, et al. Neutrophil signaling during myocardial infarction wound repair. Cell Signal. 2021;77:109816. 10.1016/j.cellsig.2020.109816 PMC7718402 33122000

[65] Schreiner D, King CG. CD4 + Memory T cells at home in the tissue: mechanisms for health and disease. Front Immunol. 2018;9:2394. 30386342 10.3389/fimmu.2018.02394 PMC6198086

[66] Lin ZL, Liu YC, Gao YL, Chen XS, Wang CL, Shou ST, et al. S100A9 and SOCS3 as diagnostic biomarkers of acute myocardial infarction and their association with immune infiltration. Genes Genet Syst. 2022;97:67–79. 35675985 10.1266/ggs.21-00073

[67] Zhu X, Yin T, Zhang T, Zhu Q, Lu X, Wang L, et al. Identification of immune-related genes in patients with acute myocardial infarction using machine learning methods. J Inflamm Res. 2022;15:3305–21. 35692951 10.2147/JIR.S360498 PMC9174022

[68] Gregoire S, Li G, Sturzu AC, Schwartz RJ, Wu SM. YY1 expression is sufficient for the maintenance of cardiac progenitor cell state. Stem Cells. 2017;35:1913–23. 28580685 10.1002/stem.2646 PMC6048588

[69] Hu J, Huang CX, Rao PP, Zhou JP, Wang X, Tang L, et al. Inhibition of microRNA-155 attenuates sympathetic neural remodeling following myocardial infarction via reducing M1 macrophage polarization and inflammatory responses in mice. Eur J Pharmacol. 2019;851:122–32. 30721702 10.1016/j.ejphar.2019.02.001

[70] Sun Y, Pinto C, Camus S, Duval V, Alayrac P, Zlatanova I, et al. Splenic marginal zone B lymphocytes regulate cardiac remodeling after acute myocardial infarction in mice. J Am Coll Cardiol. 2022;79:632–47. 35177192 10.1016/j.jacc.2021.11.051

[71] Volodarsky I, Shimoni S, Haberman D, Mirkin V, Fabrikant Y, Yoskovich Mashriki T, et al. Circulating regulatory B-Lymphocytes in patients with acute myocardial infarction: A pilot study. J Cardiovasc Dev Dis. 2022;10. 10.3390/jcdd10010002 PMC9865555 36661897

[72] Casarotti ACA, Teixeira D, Longo-Maugeri IM, Ishimura ME, Coste MER, Bianco HT, et al. Role of B lymphocytes in the infarcted mass in patients with acute myocardial infarction. Biosci Rep. 2021;41. 10.1042/BSR20203413 PMC7859321 33495783

[73] Li Y, Zhang Y, Wen M, Zhang J, Zhao X, Zhao Y, et al. Ginkgo biloba extract prevents acute myocardial infarction and suppresses the inflammation–and apoptosis–regulating p38 mitogen–activated protein kinases, nuclear factor–κB and B–cell lymphoma 2 signaling pathways. Mol Med Rep. 2017;16:3657–63. 28713946 10.3892/mmr.2017.6999

[74] Mo F, Luo Y, Yan Y, Li J, Lai S, Wu W. Are activated B cells involved in the process of myocardial fibrosis after acute myocardial infarction? An in vivo experiment. BMC Cardiovasc Disord. 2021;21:5. 33407160 10.1186/s12872-020-01775-9 PMC7789158

[75] Xu Y, Jiang K, Chen F, Qian J, Wang D, Wu Y, et al. Bone marrow-derived Naïve B lymphocytes improve heart function after myocardial infarction: a novel cardioprotective mechanism for empagliflozin. Basic Res Cardiol. 2022;117:47. 36171393 10.1007/s00395-022-00956-1

[76] Peet C, Ivetic A, Bromage DI, Shah AM. Cardiac monocytes and macrophages after myocardial infarction. Cardiovasc Res. 2020;116:1101–12. 31841135 10.1093/cvr/cvz336 PMC7177720

[77] Rizzo G, Gropper J, Piollet M, Vafadarnejad E, Rizakou A, Bandi SR, et al. Dynamics of monocyte-derived macrophage diversity in experimental myocardial infarction. Cardiovasc Res. 2023;119:772–85. 35950218 10.1093/cvr/cvac113 PMC10153424

[78] Dong Z, Hou L, Luo W, Pan LH, Li X, Tan HP, et al. Myocardial infarction drives trained immunity of monocytes, accelerating atherosclerosis. Eur Heart J. 2024;45:669–84. 38085922 10.1093/eurheartj/ehad787

[79] Dutta P, Nahrendorf M. Monocytes in myocardial infarction. Arterioscler Thromb Vasc Biol. 2015;35:1066–70. 25792449 10.1161/ATVBAHA.114.304652 PMC4409536

[80] Fraccarollo D, Neuser J, Möller J, Riehle C, Galuppo P, Bauersachs J. Expansion of CD10(neg) neutrophils and CD14(+)HLA-DR(neg/low) monocytes driving proinflammatory responses in patients with acute myocardial infarction. Elife. 2021;10. 10.7554/eLife.66808 PMC8324297 34289931

[81] Lin SF, Lin HA, Hou PC, Tsai HW, Hou SK. Monocyte distribution width enhances the detection of infection in patients after primary percutaneous coronary intervention. PLoS ONE. 2025;20:e0325314. 40460177 10.1371/journal.pone.0325314 PMC12132996

[82] Bi FF, Cao M, Pan QM, Jing ZH, Lv LF, Liu F, et al. ITFG2, an immune-modulatory protein, targets ATP 5b to maintain mitochondrial function in myocardial infarction. Biochem Pharmacol. 2024;226:116338. 38848780 10.1016/j.bcp.2024.116338

[83] Carpenter SM, Lu LL, Leveraging Antibody B. Cell and Fc receptor interactions to understand heterogeneous immune responses in tuberculosis. Front Immunol. 2022;13:830482. 35371092 10.3389/fimmu.2022.830482 PMC8968866

[84] Jones PW, Mallat Z, Nus M. T-Cell/B-Cell interactions in atherosclerosis. Arterioscler Thromb Vasc Biol. 2024;44:1502–11. 38813700 10.1161/ATVBAHA.124.319845 PMC11208060

[85] Sundararaj S, Casarotto MG. Molecular interactions of IRF4 in B cell development and malignancies. Biophys Rev. 2021;13:1219–27. 35059038 10.1007/s12551-021-00825-6 PMC8724474

