# Supplementary figures and images for: Identification and validation of ANXA3 and SOCS3 as biomarkers for acute myocardial infarction related to sphingolipid metabolism
Source: Hereditas. 2025 Aug 4;162:150. doi: 10.1186/s41065-025-00515-3 (PMC12323171; doi:10.1186/s41065-025-00515-3)

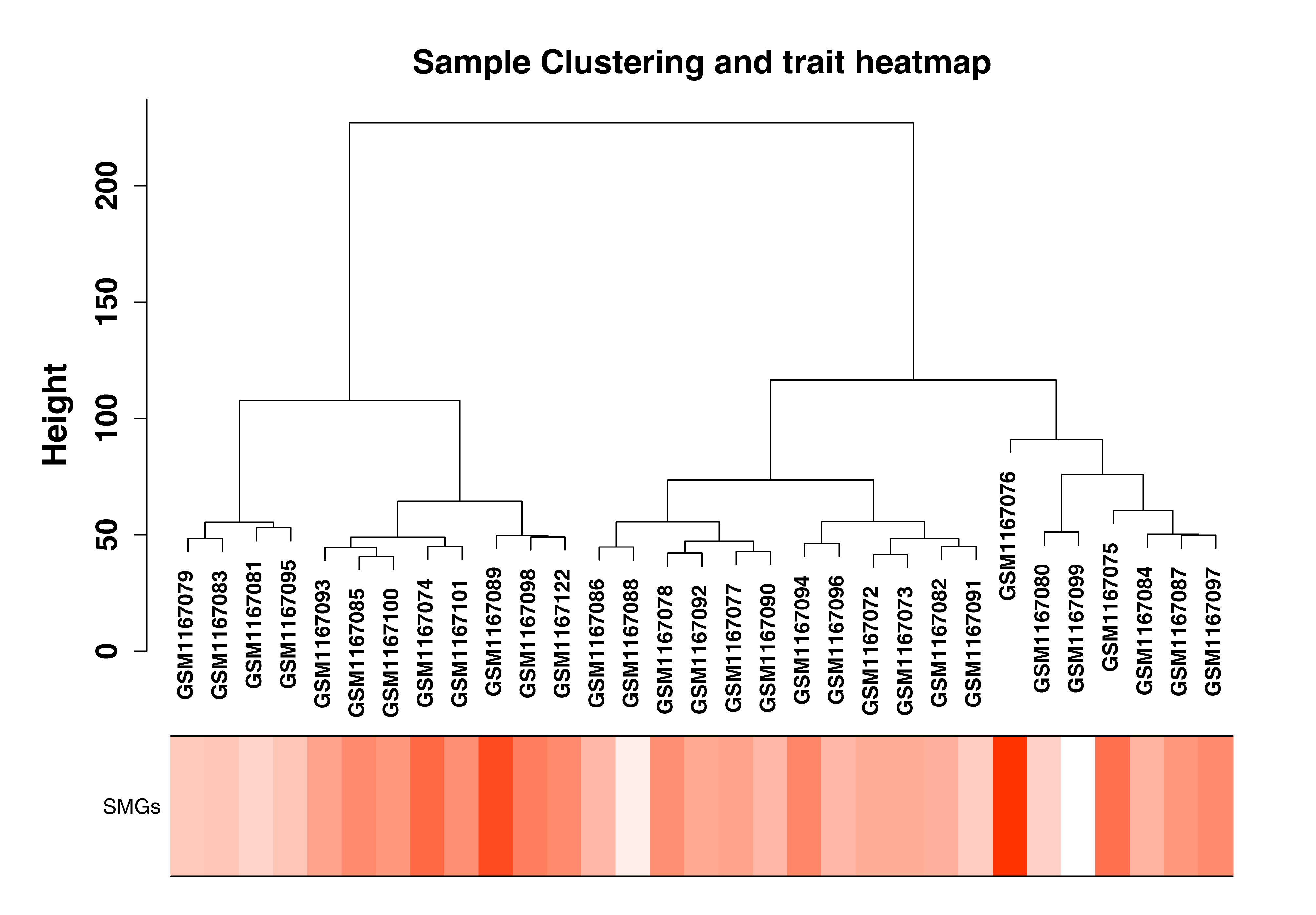

Supplement: Supplementary file 2 — Supplementary Material 2 [file 41065_2025_515_MOESM2_ESM.tif]

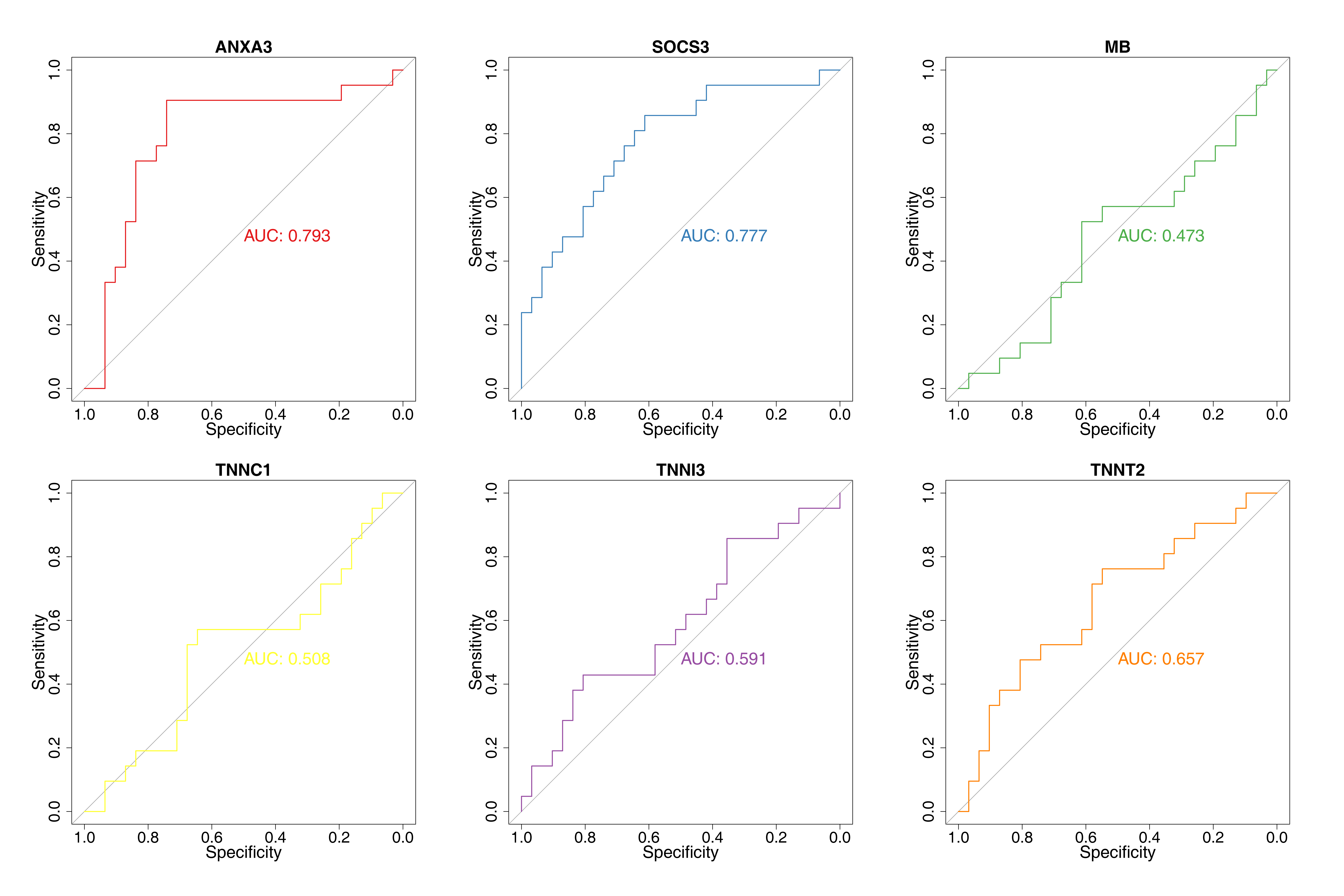

Supplement: Supplementary file 3 — Supplementary Material 3 [file 41065_2025_515_MOESM3_ESM.tif]

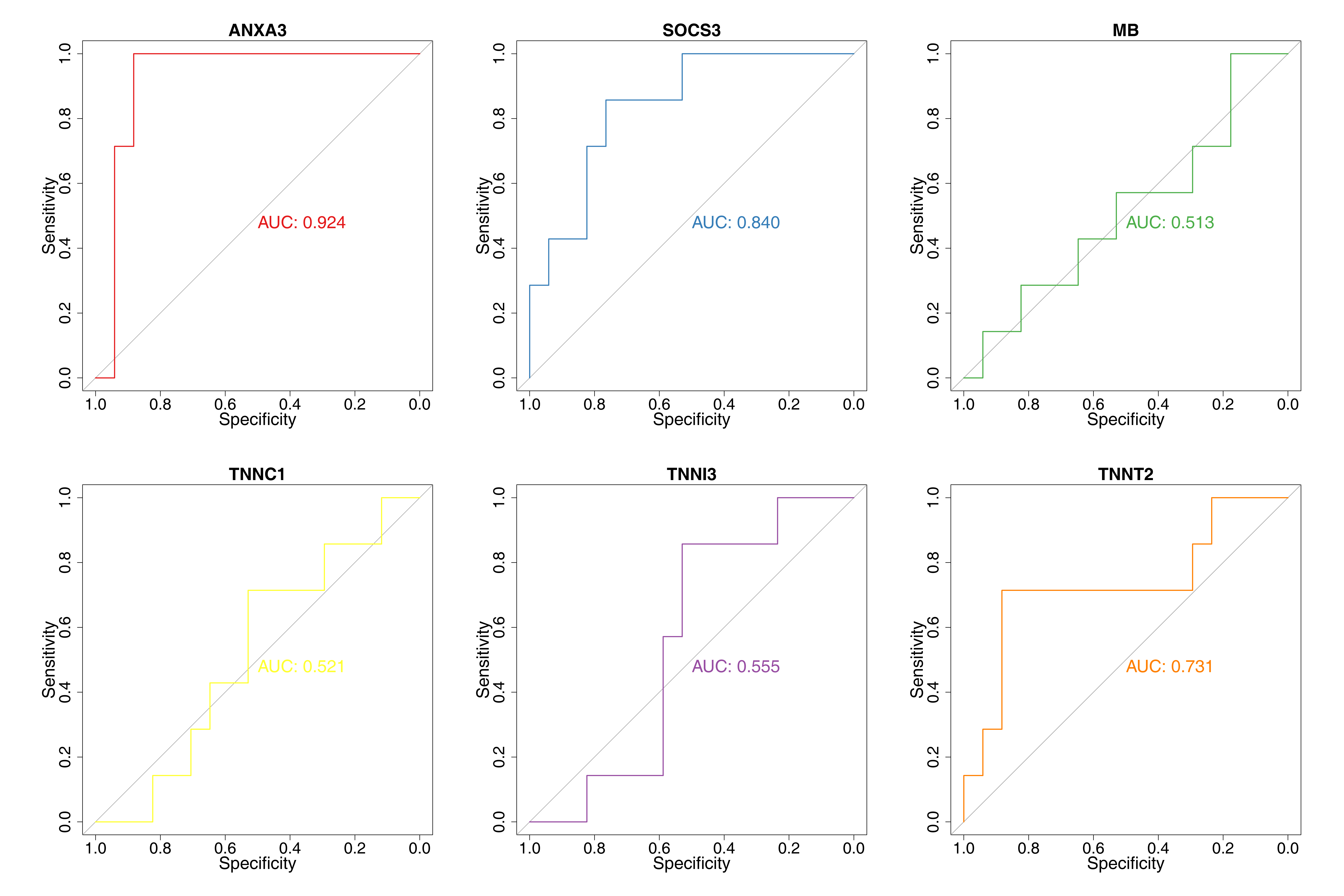

Supplement: Supplementary file 4 — Supplementary Material 4 [file 41065_2025_515_MOESM4_ESM.tif]

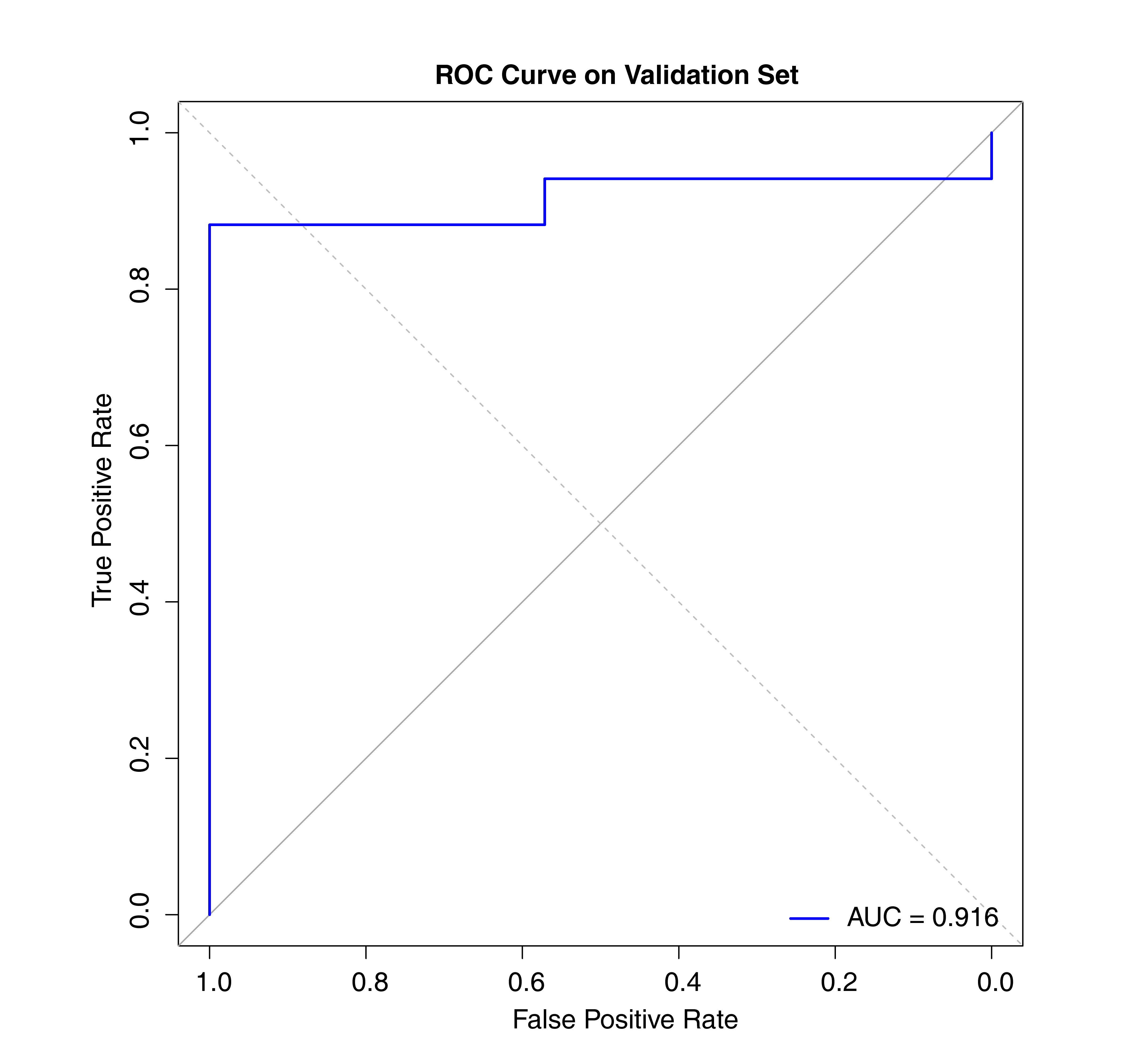

Supplement: Supplementary file 5 — Supplementary Material 5 [file 41065_2025_515_MOESM5_ESM.tif]
